# Analysis of weighted co-regulatory networks in maize provides insights into new genes and regulatory mechanisms related to inositol phosphate metabolism

**DOI:** 10.1186/s12864-016-2476-x

**Published:** 2016-02-24

**Authors:** Shaojun Zhang, Wenzhu Yang, Qianqian Zhao, Xiaojin Zhou, Ling Jiang, Shuai Ma, Xiaoqing Liu, Ye Li, Chunyi Zhang, Yunliu Fan, Rumei Chen

**Affiliations:** Biotechnology Research Institute, Chinese Academy of Agricultural Sciences, 100081 Beijing, China; National Key Facility for Crop Gene Resources and Genetic Improvement (NFCRI), 100081 Beijing, China

**Keywords:** Inositol phosphates, Metabolism, Co-regulatory network, WGCNA, Candidate gene

## Abstract

**Background:**

*D*-myo-inositol phosphates (IPs) are a series of phosphate esters. *Myo*-inositol hexakisphosphate (phytic acid, IP6) is the most abundant IP and has negative effects on animal and human nutrition. IPs play important roles in plant development, stress responses, and signal transduction. However, the metabolic pathways and possible regulatory mechanisms of IPs in maize are unclear. In this study, the B73 (high in phytic acid) and Qi319 (low in phytic acid) lines were selected for RNA-Seq analysis from 427 inbred lines based on a screening of IP levels. By integrating the metabolite data with the RNA-Seq data at three different kernel developmental stages (12, 21 and 30 days after pollination), co-regulatory networks were constructed to explore IP metabolism and its interactions with other pathways.

**Results:**

Differentially expressed gene analyses showed that the expression of *MIPS* and *ITPK* was related to differences in IP metabolism in Qi319 and B73. Moreover, WRKY and ethylene-responsive transcription factors (TFs) were common among the differentially expressed TFs, and are likely to be involved in the regulation of IP metabolism.

Six co-regulatory networks were constructed, and three were chosen for further analysis. Based on network analyses, we proposed that the GA pathway interacts with the IP pathway through the ubiquitination pathway, and that Ca^2+^ signaling functions as a bridge between IPs and other pathways. IP pools were found to be transported by specific ATP-binding cassette (ABC) transporters. Finally, three candidate genes (*Mf3*, *DH2* and *CB5*) were identified and validated using *Arabidopsis* lines with mutations in orthologous genes or RNA interference (RNAi)-transgenic maize lines. Some mutant or RNAi lines exhibited seeds with a low-phytic-acid phenotype, indicating perturbation of IP metabolism. *Mf3* likely encodes an enzyme involved in IP synthesis, *DH2* encodes a transporter responsible for IP transport across organs and *CB5* encodes a transporter involved in IP co-transport into vesicles.

**Conclusions:**

This study provides new insights into IP metabolism and regulation, and facilitates our development of a better understanding of the functions of IPs and how they interact with other pathways involved in plant development and stress responses. Three new genes were discovered and preliminarily validated, thereby increasing our knowledge of IP metabolism.

**Electronic supplementary material:**

The online version of this article (doi:10.1186/s12864-016-2476-x) contains supplementary material, which is available to authorized users.

## Background

Inositol phosphates (IPs) are a series of phosphate esters of *myo*-inositol (Additional file 1: Figure S1). IPs are abundant in the natural environment and have multiple biological functions [1]. IP3 [2] and other inositol polyphosphates—including IP6 [3], IP7 [4] and IP8 [1]—are important messengers. IP6 also plays important roles in RNA editing [5], transcription [6], DNA repair [7] and the stress response [8]. In humans, IP6 showed significant inhibition and regulation of cancer cell growth [9]. IP5 functions as a ligand in the COI1-JAZ signaling pathway [10] or as a substrate for the production of other secondary metabolites, including components of the cell wall [11] and raffinose [12].

Grain crops store excess phosphate as a single compound, inositol hexaphosphate (IP6), also known as phytic acid, which accounts for ~65–85 % of the total phosphate in grain crop seeds [13]. IP6 has been reported to be an inhibitor of the absorption of mineral nutrients such as zinc and iron [14] by animals including humans because of a lack of phytase in their digestive system [15]. Moreover, the excrement of grain-fed livestock containing IP6 can lead to phosphorus pollution [16].

The reduction of phytic acid content is a major goal of plant-breeding programs that aim to improve the nutritional quality of crops. Efforts to breed low-phytic-acid (lpa) crops have focused on the genes involved in IP metabolism and compartmentation [17–21], while the regulatory pathways for IP synthesis and accumulation have received scant attention [22, 23]. Moreover, although high IP6 content in seeds was thought to be unnecessary for plant development [24], many *lpa* mutants showed a lethal phenotype [25] or recessionary agronomic traits [26], including reduced yield and reduced resistance to stress. However, the molecular mechanism(s) underlying these phenotypes of *lpa* plants, and the roles played by IPs in plant physiology remain unclear, owing to a generally poor understanding of the synthesis and biological functions of IPs in plants [27]. Thus, further research into the functions of IPs is warranted.

The synthesis of IPs involves sequential phosphorylation of the inositol ring [28]. Plants have two main IP synthesis pathways (Additional file 1: Figure S2). The first is the lipid-dependent pathway, which is the main source of the secondary messenger Ins(1,4,5)P_3_. IP_3_ is then phosphorylated by inositol phosphate multikinase to form IP6. The second pathway is lipid-independent and is considered the main source of IP6 in seeds. Several steps in this pathway are unknown, e.g., no genes or proteins have been identified as responsible for the conversion of IP1 into IP2. IP metabolism in maize is poorly understood. Three genes were identified as responsible for IP metabolism in maize using a reverse-genetics technique. A maize *lpa2* mutant was created by mutation of the *ZmIpk* gene. *ZmIpk* encodes an inositol phosphate kinase and is homologous with *Arabidopsis* Ins(1, 3, 4)P_3_ 5/6-kinase [17]. The maize line *lpa3* is a *Mu*-insertion mutant in which an increased level of *myo*-inositol and decreased levels of other IPs were caused by a mutation in *MIK*, which encodes a pfkB carbohydrate kinase-family protein named *myo*-inositol kinase. This protein catalyzes the production of *myo*-inositol monophosphate from *myo*-inositol and ATP [29]. Another low-phytic-acid maize mutant, termed *lpa1*, harbored a mutation in a multidrug resistance-associated protein (MRP) ATP-binding cassette (ABC) transporter, the exact molecular function of which remains unclear [19, 25]. Such findings increase our understanding of IP metabolism. However, further information regarding the metabolic pathways and molecular functions of IPs is needed.

Some genes involved in IP metabolism are present as more than one copy or as multiple splice variants. Interestingly, only one of those copies or splice variants is important for IP accumulation in seeds [18, 30], while others play roles in physical development, e.g., the gene encoding *myo*-inositol 1-phosphate synthase (*MIPS*) is required for the suppression of cell death [31]. Other than participating in the metabolism of IPs, some genes encode multifunctional proteins involved in the regulation of complex physiological processes [26, 32], e.g., *MIPS*-silenced soybean lines show inhibited seed development [20]. Thus, it is necessary to explore the regulation of IP metabolism and establish the link between IPs and these important physiological processes.

Systems biology approaches are powerful tools for exploring the links between co-regulated genes and metabolites. Metabolite abundance is controlled by gene expression and enzyme activities. Thus metabolite data can be combined with those of gene expression by means of correlation and other distance analyses [33]. Moreover, the coordinated expression pattern of a set of genes indicates the functional links among them and genes associated with the same metabolic pathway are likely to be co-expressed [34]. The network method facilitates exploration of candidate genes and the regulatory mechanisms of a pathway [35]. Modules [33, 36], which contain information regarding which biological processes are related to the candidate genes or metabolites, can be extracted from a co-expression network.

Thus, construction of a co-expression network of genes related to IPs has two advantages: first, complete coverage of all expressed functional genes, which provides information on those related to both metabolism and regulation; and second, identification of relationships between IP metabolism and other biological pathways. Weighted gene co-expression network analysis (WGCNA) [37] is more effective than other network construction methods, and weighted networks are more robust and accurate than un-weighted networks [38].

In this study, we constructed gene co-expression networks related to each IP by integrating transcriptome and metabolite data to explore the regulatory mechanism of IP metabolism. Key nodes between IP metabolism and other biological pathways were implied from the network. Three candidate genes were also predicted based on the gene co-expression network and validated by biological experiments.

## Results and discussion

### Determination and analysis of IP levels in maize seeds

#### IP6 screening in a maize germplasm collection

We screened an inbred maize collection [39] to select inbred lines with significant differences in phytic acid content. Four hundred twenty-five content values were obtained after removing abnormal values. The average content of phytic acid phosphors (PAP, phosphate in phytic acid) in this inbred maize collection was 3.9 mg/g, slightly higher than the 3.0 mg/g reported previously [13]. Ultimately, 24 line-pairs with stable IP6 content ratios (~2 times) were obtained (Methods, Additional file 2: Table S1). The PAP content of low phytic acid (LPA) lines ranged from 2.4 mg/g to 2.5 mg/g, and that of high phytic acid (HPA) lines from 4.0 to 5.4 mg/g (Additional file 1: Figure S3). B73 (HPA), Lv28 (HPA), Qi319 (LPA), and CIMBL141 (LPA) were selected for further monitoring of dynamic changes in IP levels because they were elite inbred lines used for breeding and genetic research, and the IP6 content ratios in their seeds was ≥ 2.

#### Dynamic changes in IP levels in maize kernels at different developmental stages

To explore the dynamic changes and distributions of the various IPs, we collected whole fresh seeds and embryos of maize at different developmental stages (6, 12, 18, 21, 24, and 30 days after pollination, DAP) and determined their IP levels by LC-MS/MS. We first examined each IP in the whole seeds of four inbred lines—B73 (HPA), Lv28 (HPA), CIMBL141 (LPA) and Qi319 (LPA)—to explore the dynamic changes in IP levels as a function of developmental stage.

**Fig. 1:**
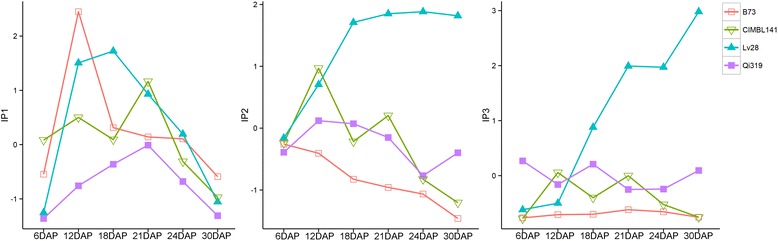
Dynamic changes in IP1 (*left*), IP2 (*middle*) and IP3 (*right*) in the seeds of four maize inbred lines. DAP: days after pollination. Data for IP1, IP2 and IP3 were normalized and plotted in R to assess the trends in IP levels. Maize inbred lines: B73 (*red*), CIMBL141 (*green*), Lv28 (*blue*), and Qi319 (*violet*)

IP4–IP6 levels were undetectable or very low in whole seeds at earlier developmental stages. Thus, we next compared the IP1–IP3 levels in seeds at the aforementioned developmental stages (Fig. 1). The results showed that IP1 displayed similar accumulation trends in each inbred line, with peaks at 12-21DAP. Moreover, the peak value of IP1 in B73 (12DAP) was 9 days earlier than in Qi319 (21DAP). IP3 showed a steady trend in B73, but underwent significant fluctuations in CIMBL141 and Qi319. IP2 levels declined continuously in B73, but in Qi319 peaked at 12DAP. It is also worth noting that IP2 and IP3 showed a sustained increasing trend in Lv28 (Fig. 1).

**Fig. 2:**
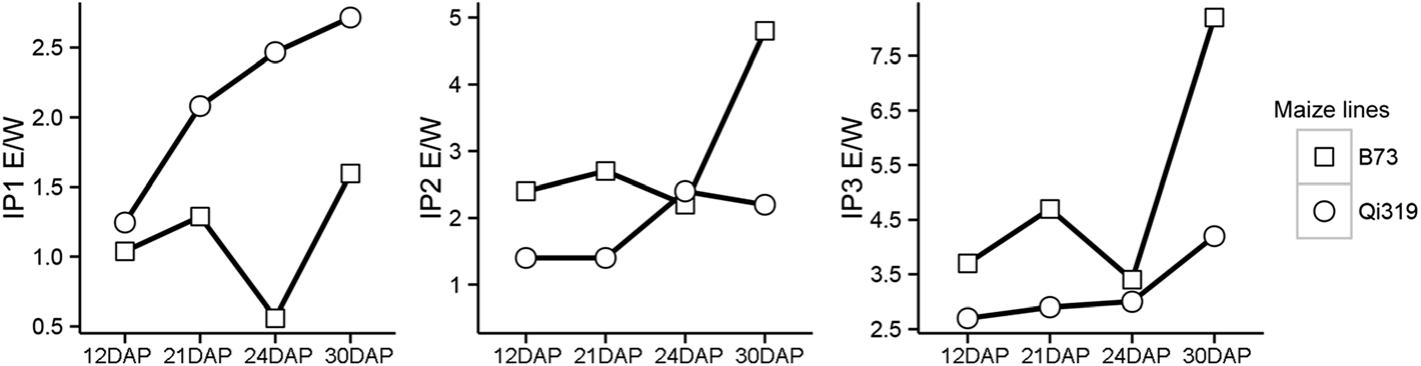
Relative abundance of IP1 (*left*), IP2 (*middle*) and IP3 (*right*) in the embryo and whole kernels of B73 and Qi319. DAP: days after pollination. E/W, ratio of IP content in the embryo (E) versus that in the whole kernel (W). E/W > 1 indicates that the IP is mainly accumulated in the embryo

**Fig. 3:**
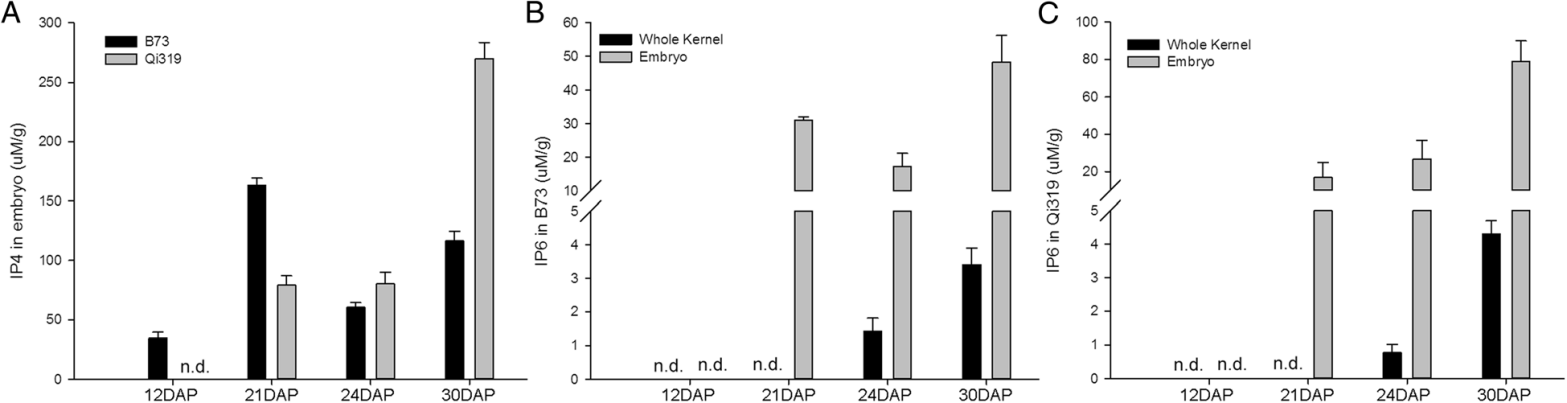
Distribution and abundance of IP4 and IP6 in the embryo and whole seeds of B73 and Qi319. DAP: days after pollination. **a** IP4 abundance in the embryos of B73 and Qi319. **b** IP6 distribution comparing the embryo with the whole kernel of B73. **c** IP6 distribution comparing the embryo with the whole kernel of Qi319. n.d., not detected

We next determined IP levels in embryos (E) and compared them with the IP levels in whole seeds (W) of B73 and Qi319. The results showed that IPs accumulated mainly in embryos, as indicated by content ratios considerably greater than 1 (E/W > 1, Fig. 2). Moreover, IPs with higher phosphate numbers—such as IP4 and IP6—were not detected before 21DAP in whole seeds (Fig. 3). For example, in contrast with B73, IP4 was not detected before 21DAP in Qi319 (Fig. 3a); IP6 was first detectable in embryos at 21DAP, and its levels were significantly higher in embryos than in the whole kernels (Fig. 3b, c). Interestingly, in the fresh samples, the IP6 level in B73 was slightly lower than that in Qi319, despite B73 being a high-phytic-acid content line. IP5 was detected in all embryos but its levels were significantly lower at 21DAP in B73 and Qi319 (Additional file 1: Figure S4), which may be related to the abundances of IP4 and IP6.

In summary, our data showed that IPs accumulated mainly in embryos, and the fact that IP4 and IP6 were not detected before 21DAP in whole seeds (Fig. 3) suggests that phytic acid was synthesized sequentially in the embryo, and that the stages of IP synthesis are associated with different developmental stages of the embryo. In maize, the compartment in which phytic acid is synthesized has been reported [29], however, our data confirmed that, at the metabolite level, IP6 accumulates mainly in the embryo.

Taking into consideration the abundance and distribution of each IP, these results indicated clear sampling points for RNA-Seq analysis. We therefore subjected embryos of B73 and Qi319 (Lv28 and CIMBL141 were not selected because they were not growing well in the field, and were susceptible to diseases and insect pests) at 12, 21 and 30DAP to RNA-Seq and microRNA-Seq analyses to explore the regulation of IP metabolism. Data-mining workflows are shown in Additional file 1: Figure S5.

### Transcriptome analysis of B73 and Qi319

#### Alternative splicing and differentially expressed gene (DEG) analysis

To further understand the regulation of IP metabolism, we performed RNA-Seq analyses of maize embryos at different developmental stages. To verify that the gene expression patterns were consistent with previous reports, we determined the expression levels of three known genes—*MIK* (GRMZM2G361593) [29], *ITPK-1* (GRMZM2G456626) [17] and *ABC transporter* (GRMZM5G820122) [19]—by *q*RT-PCR (Additional file 1: Figure S6). *MIK* expression in Qi319 peaked at about 21DAP, similar to the findings of Shi et al*.* [29]. In contrast, *MIK* expression in B73 declined continuously. The expression levels of *ITPK-1* and *ABC transporter* were similar to that of *MIK*, but exhibited different expression patterns. Moreover, the greater expression of the three genes in B73 at 12DAP is likely related to the earlier detection of IP4 (Additional file 1: Figure S6, Fig. 3a).

Alternative splicing (AS) plays important roles in gene functions [40] and is the main source of protein diversity [41]. For instance, *OsLpa1* has three splices in rice, but only the longest transcript is responsible for the phytic acid content of seeds [18]. IP metabolism genes also have specific AS modes (Additional file 1: Figure S7). The gene encoding inositol 1,4,5-triphosphate 5-phosphatase (EC 3.1.3.56) (GRMZM2G004301) has splice sites at the first exon (alternative first exon, AFE) and the last exon (alternative last exon, ALE). This AS mode was present in B73 but not in Qi319. In B73, *ITPK-2* (GRMZM2G179473) exhibited two variants: 3' alternative splicing (alternative 3' splice site, 3AS) and exon skipping (ES) at 12 and 30DAP, while at 21DAP, only 3AS was present. Interestingly, only 3AS of *ITPK-2* was detected by RNA-Seq at 30DAP in Qi319. Therefore, the variation in AS modes observed among the inbred lines at different developmental stages is likely related to the metabolic differences between B73 and Qi319 (Additional file 1: Figure S7).

The different abundances of each IP among plant tissues would be due to differences in the expression levels of the corresponding genes. To gain an increased understanding of the differences in IP metabolism, we compared gene expression levels between the B73 and Qi319 lines. Genes with fold changes (FCs) > 2 and FDR < 0.01 were considered to be DEGs.

In general, a greater number of DEGs was found among the genetic backgrounds, rather than the developmental stages. In total, 1481 genes were differentially expressed in the embryos at all time points (Additional file 1: Figure S8). Gene ontology (GO) annotations of the DEGs indicated that they were enriched in the “metabolic process”, “binding”, “catalytic activity” and “transporter activity” categories (Additional file 1: Figure S9A-C). Cluster of Orthologous Groups of proteins (COG) annotation showed that ~500 DEGs were enriched in the “Carbohydrate transport and metabolism”, “Transcription” and “Signal transduction mechanism” categories (Additional file 1: Figure S9E-F).

We also mapped the DEGs to KEGG pathways (Entry: map00562, *p* < 0.05, multiple hypothesis-based testing) (Additional file 1: Figure S10). *MIPS* and *ITPK-1* were differentially expressed in Qi319 and B73 at all time points. *MIPS* (EC 5.5.1.4) catalyzes the conversion of glucose-6-phosphate (G6P) to *myo*-inositol 3-phosphate (IP1), the first step in IP synthesis (Additional file 1: Figure S10A-C). The differential expression of *MIPS* implied that the metabolic differences between B73 and Qi319 began at the first step of IP synthesis. *ITPK-1* catalyzes the conversion of IP3 to IP4 and was upregulated at 12DAP but downregulated at 21DAP and 30DAP in B73 (Additional file 1: Figure S10A-C), which was consistent with the *q*RT-PCR results (Additional file 1: Figure S6). This expression pattern was likely related to the appearance of IP4 in the embryo (Fig. 3a). In addition, some inositol transporter genes and inositol triphosphate 5-phosphase genes were also differentially expressed in B73 and Qi319 (Additional file 1: Figure S11).

#### Differentially expressed transcription factors and microRNAs

To our knowledge, no transcription factors (TFs) involved in the regulation of IP metabolism have been reported to date. We extracted 303 TF genes from the DEGs (Additional file 3), including 13 novel assembled genes.

Some of the differentially expressed TFs were consistently up- or down-regulated in B73 or differentially expressed at different developmental stages (Additional file 1: Figure S12A). Those TFs were enriched in four families according to their molecular functions (Additional file 1: Figure S12B)—WRKY, MYB, bHLH and ethylene-responsive TFs. WRKY family genes are actively involved in abiotic/biotic stress and hormone responses, e.g.*,* gibberellic acid, GA [42] and ~30 WRKY genes were persistently differentially expressed during all developmental stages assessed in this study. MYB and bHLH domain-containing TFs are associated with the regulation of plant development, the stress response, and temporal regulation of gene expression [43]. Twenty MYB genes were sustainably differentially expressed. Ethylene-responsive TFs are involved in primary and secondary metabolite regulation, and were (40 genes) differentially expressed mainly at 12DAP (Additional file 1: Figure S12B). These results provide resources for further research into IP related TFs.

MicroRNA-Seq analyses showed that five differentially expressed microRNAs in B73 and Qi319 targeted IP-related genes (according to the GO annotation and microRNA target prediction, Table 1). Three of these candidate microRNAs had predicted stem-loop structures, and their expression tended to be negatively correlated (e.g., GRMZM2G135978 was negatively correlated with zma-miR393c-5p, Pearson coefficient = −0.67) with that of their target genes (Additional file 1: Figure S13).

**Table 1:** Candidate microRNAs and their predicted target genes

Gene ID	microRNA	GO annotation of target gene
GRMZM2G085568	2_5358452	Type I inositol 1,4,5-trisphosphate 5-phosphatase 12
GRMZM2G085568	4_5805098	
GRMZM2G085568	7_2680918	
GRMZM2G108767	2_5300476	1-phosphatidylinositol-3-phosphate 5-kinase FAB1A
GRMZM2G135978	zma-miR393c-5p	Transport inhibitor response 1-like protein, inositol hexakisphosphate binding

### Weighted gene co-expression network construction and analysis

The abundance of each IP in B73 and Qi319 embryos showed similar patterns with the expression of known genes (Additional file 1: Figure S4, Figure S6). To explore the regulation of IP metabolism and discover new genes involved in IPs, we first constructed gene co-expression networks weighted with metabolite data (WGCNA method, Additional file 1: Figure S15). Ultimately, six IP-related gene co-regulation modules (correlation coefficient > 0.8*, p <* 0.01) were extracted from the whole transcriptome (Table 2 and Additional file 1: Figure S14, Figure S15), three of which (“magenta2”, “cornsilk” and “dodgerblue4”) were subjected to further analysis. The networks were compressed using the Power Graph method [44], cutting nodes with low connectivity and merging the shared edges to form power edges. The compressed ratio of each network ranged from 75 to 92 % (Additional file 2: Table S2). The networks were decoded based on guide-genes and metadata analyses using plugins embedded in Cytoscape (see Methods).

**Table 2:** Gene co-expression modules related to each inositol phosphate

Metabolite	Module name	Correlation type	coefficient	*p*-value
IP1	dodgerblue4	Positive	0.87	3 × 10^−4^
IP2	salmon1	Negative	−0.98	4 × 10^−4^
IP2	burlywood2	Positive	0.96	3 × 10^−4^
IP3	burlywood2	Positive	0.86	3 × 10^−4^
IP4	magenta2	Negative	−0.87	2 × 10^−3^
IP5	cornsilk	Positive	0.91	1 × 10^−4^
IP6	steelblue4	Positive	0.81	2 × 10^−4^
IP6	cornsilk	Positive	0.86	3 × 10^−4^
IP6	magenta2	Negative	−0.87	3 × 10^−4^

#### NADH: ubiquinone oxidoreductase gene co-regulated with *ITPK* in the “magenta2” network

**Fig. 4:**
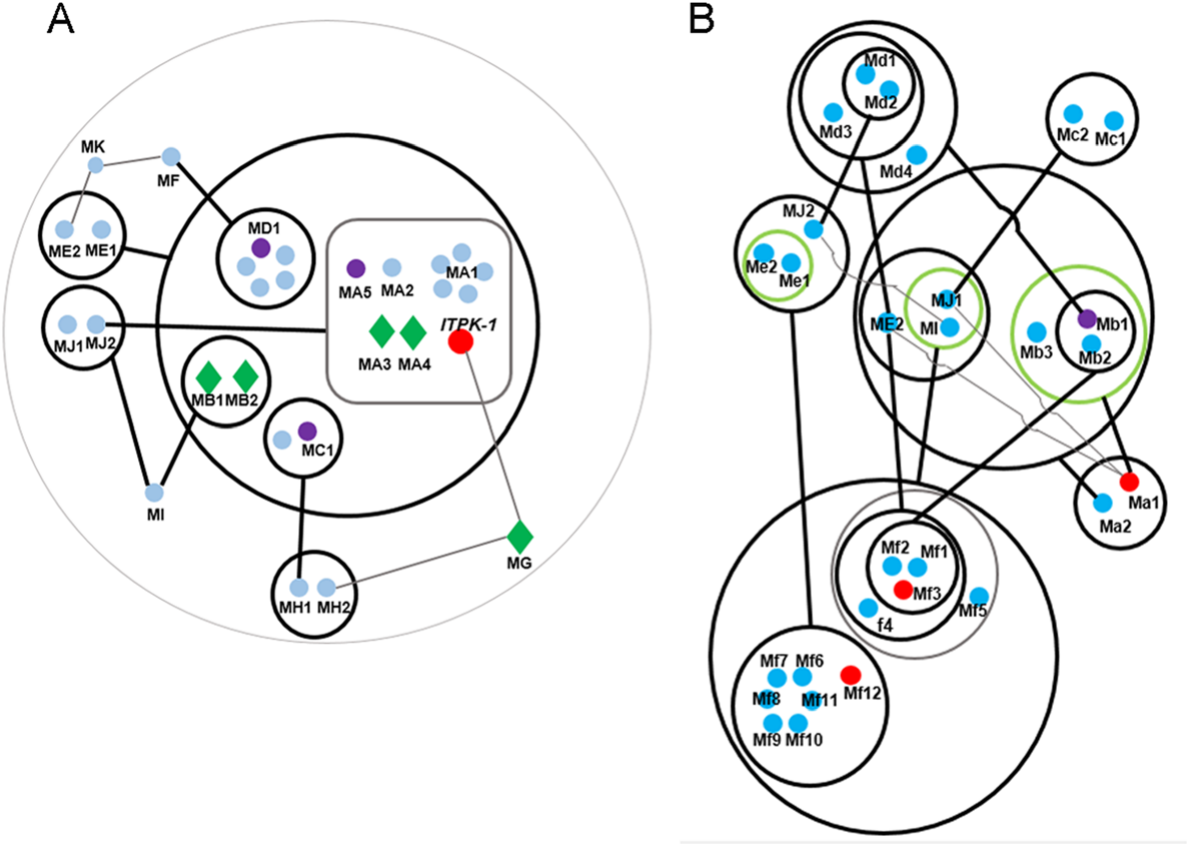
Sub-networks of “magenta2”. **a** sub-network 1 of “magenta2”. **b** sub-network 2 of “magenta2”. Thick circles are power nodes and genes in green circles are connected to each other; thick lines are power edges, and the fine gray lines are common edges. Red, nodes related to inositol phosphate or possible candidate genes; green diamonds, transcription factors; violet nodes, other inositol phosphate-related genes; and blue nodes, other genes without direct inositol phosphate-related annotation. The sub-networks were manually redrawn to clarify the nodes and edges. The original network and node annotations are shown in Additional file 4

*ITPK-1* was co-expressed with the NADH:ubiquinone oxidoreductase (EC 1.6.5.3) gene (GRMZM2G084914, node MJ2, correlation coefficient = 0.88, *p*-value = 8 × 10^−3^), metal-binding protein gene (GRMZM2G092867, node ME2, correlation coefficient = 0.59, *p*-value = 2 × 10^−3^), UDP-glycosyltransferase gene (AC199541.4_FG004, node ME1, correlation coefficient = 0.51, *p*-value = 3 × 10^−3^), and a VQ-motif (interacting with WRKY TFs) [45] -containing protein gene (AC194056.3_FG008, node MG, correlation coefficient = 0.65, *p*-value = 1 × 10^−3^) in the “magenta2” network, which was negatively correlated with IP6 (−0.87, *p*-value = 3 × 10^−4^) and IP4 (−0.87, *p*-value = 2 × 10^−3^; the sub-network 1 of “magenta2”, Fig. 4a and Additional file 4). As described above, the correlation of *ITPK-1* with the VQ-coding gene suggested a mechanism by which WRKY TFs regulate the expression of genes related to IP metabolism.

NADH:ubiquinone oxidoreductase consumes or produces NADH and functions as a key enzyme in oxidative phosphorylation and the electron transport chain [46, 47]. The relationships between IP metabolism and NADH have not been discussed previously. Although it is known that NADH is required for catalysis by MIPS of glucose 6-phosphate to form inositol monophosphate [48], why the expression of *ITPK-1* and IP4/IP6 was correlated with that of the NADH:ubiquinone oxidoreductase gene is unclear. The IP3 receptor interacted with NADH to release Ca^2+^ [49, 50], and Voronina et al*.* reported that the NADH level was related to Ca^2+^ signaling [51], which was directly modulated by the NAD^+^/NADH redox state [52]. Therefore, the correlation of *ITPK-1* expression with that of the NADH:ubiquinone oxidoreductase gene essentially reflects the co-regulation of redox homeostasis (NADH) and Ca^2+^ signaling, and implies that sequential changes in IP3 levels will in turn affect IP metabolism.

#### Interaction of the gibberellic acid signaling pathway with IP metabolism in the “magenta2” network

We extracted another sub-network from “magenta2” using a gene (GRMZM2G154565, node Ma1) annotated as “1-phosphatidylinositol 4,5-bisphosphate phosphodiesterase” (a member of the phospholipase C gene family) as a guide-gene (Fig. 4b, and Additional file 4, the sub-network 2 of “magenta2”). This network overlapped through four nodes (ME2, MI, MJ1, and MJ2) with the first sub-network mentioned above (Fig. 4a).

Phospholipase C (PLC, node Ma1) is involved in Ca^2+^ signal transduction [53, 54] and IP metabolism [55] by releasing IP3 from phosphatidylinositol. The PLC gene was correlated with node ME2 (correlation coefficient = 0.86, *p*-value = 1 × 10^−2^), which was also correlated with *ITPK-1* (Fig. 4a). This indirect interaction is consistent with the roles of PLC and *ITPK-1* in IP metabolism.

F-box protein and metal ion transporter genes lie in the regulation center between the PLC gene and the P-loop–containing protein gene (GRMZM2G123544, node Mf3). Some small molecules, e.g., auxin, can bind to F-box protein and mediate ubiquitination to regulate protein function at the posttranslational level [56, 57]. Interestingly, two gibberellic acid (GA)-related genes, i.e., node Mc1 (GRMZM2G070068) and Md1 (GRMZM2G013016), were also in contact with node Mf3 by sharing the Mb1, ME2, and MJ1 nodes, which were also in contact with the PLC gene (Fig. 4b).

The finding that the PLC gene shared nodes with GA-related genes is noteworthy. Metadata analyses showed that the GA signaling pathway has biological relationships with inositol phosphates. IP6 is hydrolyzed by phytase in the presence of GA during seed germination [58], and some *lpa* mutant crop lines show a reduced germination phenotype [27, 59]. Murthy et al*.* [60] found that phosphatidylinositol metabolism was altered by GA in the aleurone layer of barley. Microarray data analysis showed that IP metabolism genes also responded to GA and ABA induction in rice [61]. Fleet et al*.* [62] reported that Arabidopsis *5ptase* mutants were hypersensitive to paclobutrazol (a GA synthesis inhibitor), suggesting a relationship between elevated IP3 levels and decreased GA signal transduction. Therefore, rather than a direct interaction, it is reasonable that GA-related genes and IP-related genes were co-regulated by nodes Mb1 (F-box protein gene), ME2 (metal ion-binding protein gene) and MJ1 (putative phospholipase gene) in this sub-network. Thus, this network predicts the potential mechanism and key nodes of the interactions between IPs and GA.

Several other genes also contacted the guide gene by sharing nodes Mb1 and ME2, including a P-loop containing (IPR027417) protein-coding gene (GRMZM2G123544, node Mf3). A P-loop domain is also present in the Lpa1 ABC transporter [19, 63]. Node Mf12 (GRMZM2G146041) is another P-loop-containing protein-coding gene, which was further from the guide gene compared with node Mf3. Therefore, we selected Mf3 as the first candidate gene involved in IP metabolism. *Mf3* likely encodes an enzyme because it lacks a predicted transmembrane domain but contains an ATPase domain (IPR027417).

#### Carbohydrate-related transporter genes in the “cornsilk” network

**Fig. 5:**
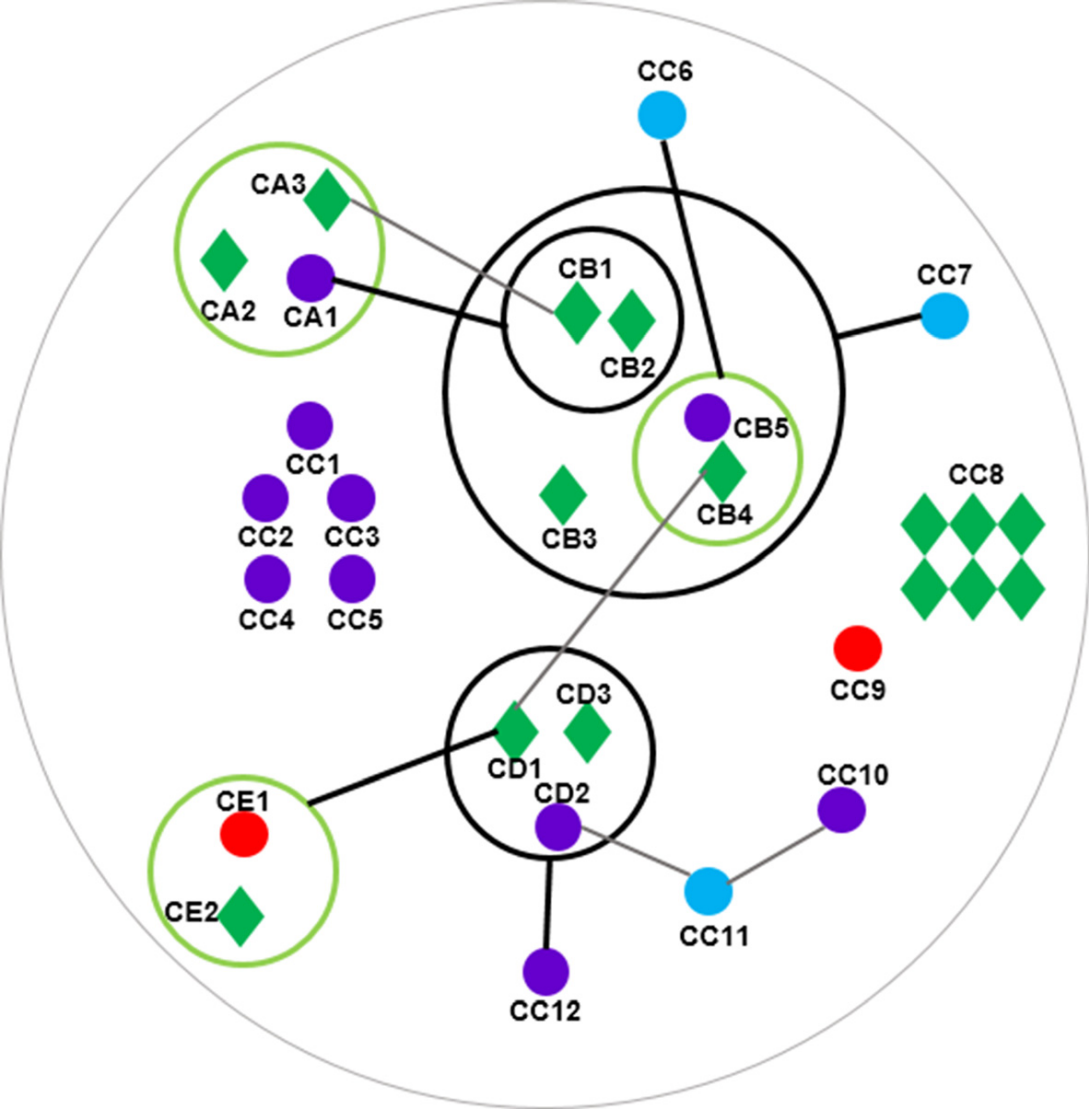
Sub-network of “cornsilk”. All symbols are as in Fig. 4, except that violet nodes indicate transporter-related genes. The original network is shown in Additional file 5

The “cornsilk” network was correlated with both IP5 (correlation coefficient = 0.91, *p*-value = 1 × 10^−4^) and IP6 (correlation coefficient = 0.86, *p*-value = 3 × 10^−4^). Interestingly, many transporter genes (10 nodes, >30 %) and transcription factor genes (15 nodes, 50 %) were in this network (Fig. 5, and Additional file 5).

Unexpectedly, the multidrug resistance-associated ATP-binding cassette (ABC) transporter gene (GRMZM5G820122, *ZmMRP4*) [19, 25] was not in this network, despite its similar expression pattern to those of *ITPK-1* and *MIK*. Many identified ABC transporters are multi-role and have various substrates, including lipids, ABA, and other hormones [64–66]. The low-phytic-acid phenotype of *lpa1* mutants or transgenic lines in rice [63], maize [19, 25, 67], and soybean [63, 68, 69] suggest that other unknown transporters are also responsible for IP transport between cells or organs. This may explain the lack of a correlation between *ZmMRP4* expression and IP6 level.

Most transporter genes in this network were related to carbohydrate metabolism. Representative genes include a UDP-galactose transporter (GRMZM2G089630, node CD2), carbohydrate/inositol-transporters (GRMZM2G063824, GRMZM2G064437, nodes CA1 and CC1), and a glycerol-3-phosphate transporter (GRMZM2G078757, node CC4).

The correlation between IP6 levels and the expression of carbohydrate transporters (correlation coefficient ranged from 0.51 to 0.95, *p*-value < 4 × 10^−3^) would imply some interesting biological relationships. Metabolite profiling of rice showed that galactose and galactinol levels were increased in *lpa* mutant lines [70]. In fact, MIPS consumes glucose 6-phosphate and NADPH to generate inositol monophosphate, producing UDP-glucose as a by-product. UDP-glucose is then converted into UDP-galactose. Therefore, the correlation between IPs and UDP-transporters would indicate the presence of metabolic cooperativity between IPs and UDP-galactose. IP6, or possibly other IPs, affects carbohydrate metabolism and composition by an unclear mechanism, e.g., several *lpa* mutants of maize exhibited decreased starch content [67]. In a study of the pleiotropic effects in the *lpa1* mutant, neither the total starch content nor the amylose/amylopectin ratio was altered, but the structure and size of granules differed from those in the wild type [71]. Thus this network illustrates the possible interactions between IPs and carbohydrate metabolism.

Raboy hypothesized that ion transporters are involved in IP6 transport to vesicles [27]. Indeed, an H^+^ transporter gene (GRMZM2G075900, node CB5) was also identified in this network. Lemtiri-Chlieh et al*.* found that ABA increased IP6 levels, and that IP6 was a potent Ca^2+^-dependent inhibitor of K^+^ traffic into guard cells [72]. IP6 accumulated mainly in vacuoles and its accumulation was enhanced when cells were grown in the presence of high concentrations of inorganic phosphates containing K^+^, Ca^2+^, or Zn^2+^ [73]. H^+^-transporters are a type of H^+^-translocating pyrophosphatase. Takasu et al*.* found that IP6 could directly interact with and inhibit H^+^-pyrophosphatase activity [74]. The H^+^-transporter (node CB5, GRMZM2G075900) was likely involved in the translocation of IP6 into vesicles and therefore *CB5* was selected as a candidate gene for further study.

#### Specific ABC transporters and IP compartmentation

**Fig. 6:**
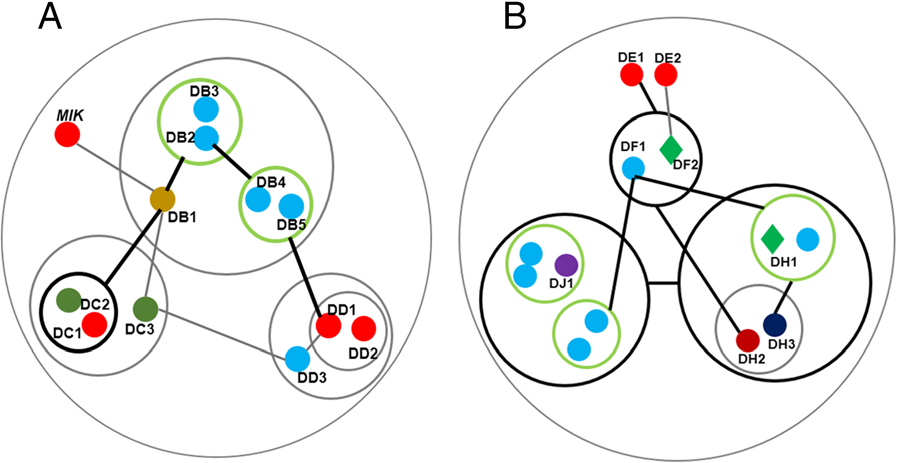
Sub-networks of “dodgerblue4”. **a** sub-network 1 of "dodgerblue4". **b** sub-network 2 of "dodgerblue4". The nodes indicated by *green circles* are ABC transporter genes and the node in *dark blue* is the *OsLpa1-like* gene. Other symbols are as in Fig. 4. The original network is shown in Additional file 6

IP1 was correlated with the “dodgerblue4” network (correlation coefficient = 0.87, *p*-value = 3 × 10^−4^), which contained the *myo*-inositol kinase (MIK) gene (GRMZM2G361593, node DA1) (Fig. 6a, Additional file 6, sub-network 1 of “dodgerblue4”). Similar to the “magenta2” network, *MIK* expression was correlated with that of an ubiquitin-related gene (GRMZM2G136313, node DB1, correlation coefficient = 0.78, *p*-value = 1 × 10^−3^). This implies that the protein ubiquitination pathway plays an important role in the regulation of IP metabolism. Two ABC transporter family genes (GRMZM2G009464, GRMZM2G072850; nodes DC2 and DC3) were also correlated with a ubiquitin-related gene (node DB1, correlation coefficients were 0.86 and 0.65 respectively, *p*-value < 1 × 10^−2^). The interaction between DB1 with the two ABC transporters (nodes DC2 and DC3) and *MIK* (node DA1) supports the hypothesis that specific transporters are responsible for the transport of inositol or inositol monophosphate to the embryo from other organs [27]. In bacteria, some ABC transporters bind specifically to inositol [75]. A soybean *Mrp1* mutant exhibited reduced seed inositol levels and altered ABA sensitivity. Moreover, its *Mrp2* paralog was unable to complement the mutant phenotype [63]. This suggests that ABC transporters play multiple roles and that specific ABC transporters are responsible for inositol/inositol phosphate transport.

The purple acid phosphatase (PAP)-like gene (GRMZM5G881649, node DJ1, Fig. 6b, Additional file 6, sub-network 2 of “dodgerblue4”) was co-expressed with the *OsLpa1-like* gene (GRMZM2G342327, node DH3, correlation coefficient = 0.90, *p*-value = 5 × 10^−3^) and an ABC transporter-like gene (GRMZM5G874955, node DH2, correlation coefficient = 0.93, *p*-value = 5 × 10^−3^). PAP is an acid phosphatase with phytase activity [76]. Overexpression of the PAP gene in *Arabidopsis* activated Ca^2+^ signaling [8], indicating a relationship between PAP and IP metabolism. Thus, the correlation between node DH2 with *OsLpa1-like* (DH3) and *PAP* (DJ1) was notable. Moreover, node DH2 was also in contact with an ethylene-responsive transcription factor (GRMZM5G830365, node DF2), which was in turn in contact with two IP-related genes (nodes DE1 and DE2). Therefore, node DH2 (GRMZM5G874955) was selected as a candidate gene for further study to confirm whether it is involved in IP transport.

Interestingly, similar to the “magenta2” network, a GA-related gene (GRMZM5G861082) was directly/indirectly in contact with that of a type C ABC transporter gene (GRMZM2G145446), a phosphatidylinositol 4-kinase gene (GRMZM2G137558), and an ABC transporter 5-like (*ZmMRP5*) gene (GRMZM2G105570). This suggested an interaction of IP metabolism or Ca^2+^ signaling with the GA pathway.

### Validation of the candidate genes

To assess the network, we selected three candidate genes for further validation. We use node names to represent each candidate gene (Table 3). According to the network information, we predicted that gene *Mf3* from “magenta2” would be involved in IP metabolism as an enzyme, gene *DH2* from “dogerblue4” would be responsible for IP transport across organs, and gene *CB5* from “cornsilk” would be related to IP co-transport into vesicles.

**Table 3:** Candidate genes selected from the gene co-expression networks

Gene code	MaizeGDB ID	From module	Annotation	Orthologous	T-DNA line
*Mf3*	GRMZM2G123544	magenta2	IPR027417	NA	NA
*CB5*	GRMZM2G075900	cornsilk	IPR004131	AT1G16780	SALK_044701
*DH2*	GRMZM5G874955	dodgerblue4	IPR027417; IPR013525	AT1G15520	SALK_013945

**Fig. 7:**
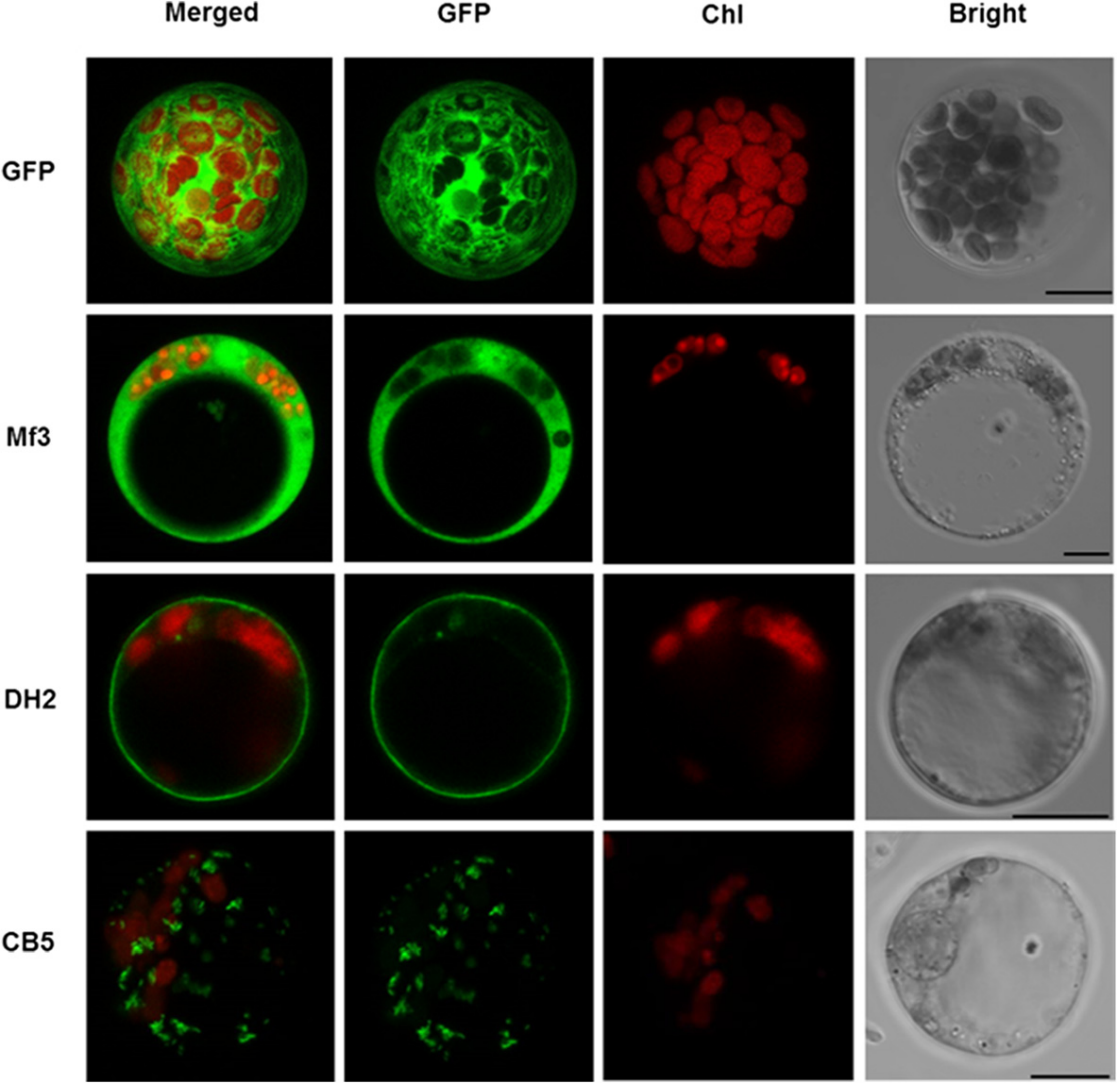
Transient transformation of maize protoplasts with GFP-fusion constructs. Bar = 10 μm. Three candidate genes: *Mf3*, GRMZM2G123544; *DH2*, GRMZM5G874955; *CB5*, GRMZM2G075900. GFP: pRTL2 vector

We analyzed the expression profiles of the three candidate genes in maize embryo (Additional file 1: Figure S16). The *Mf3* and *DH2* genes showed expression patterns similar to those of *ITPK-1* and *MIK*, respectively. GFP-fused transient transformation in maize protoplasts showed that *Mf3* was expressed in the cytoplasm, *DH2* in the plasma membrane, and *CB5* in vesicles, which is consistent with their expected molecular functions (Fig. 7).

**Fig. 8:**
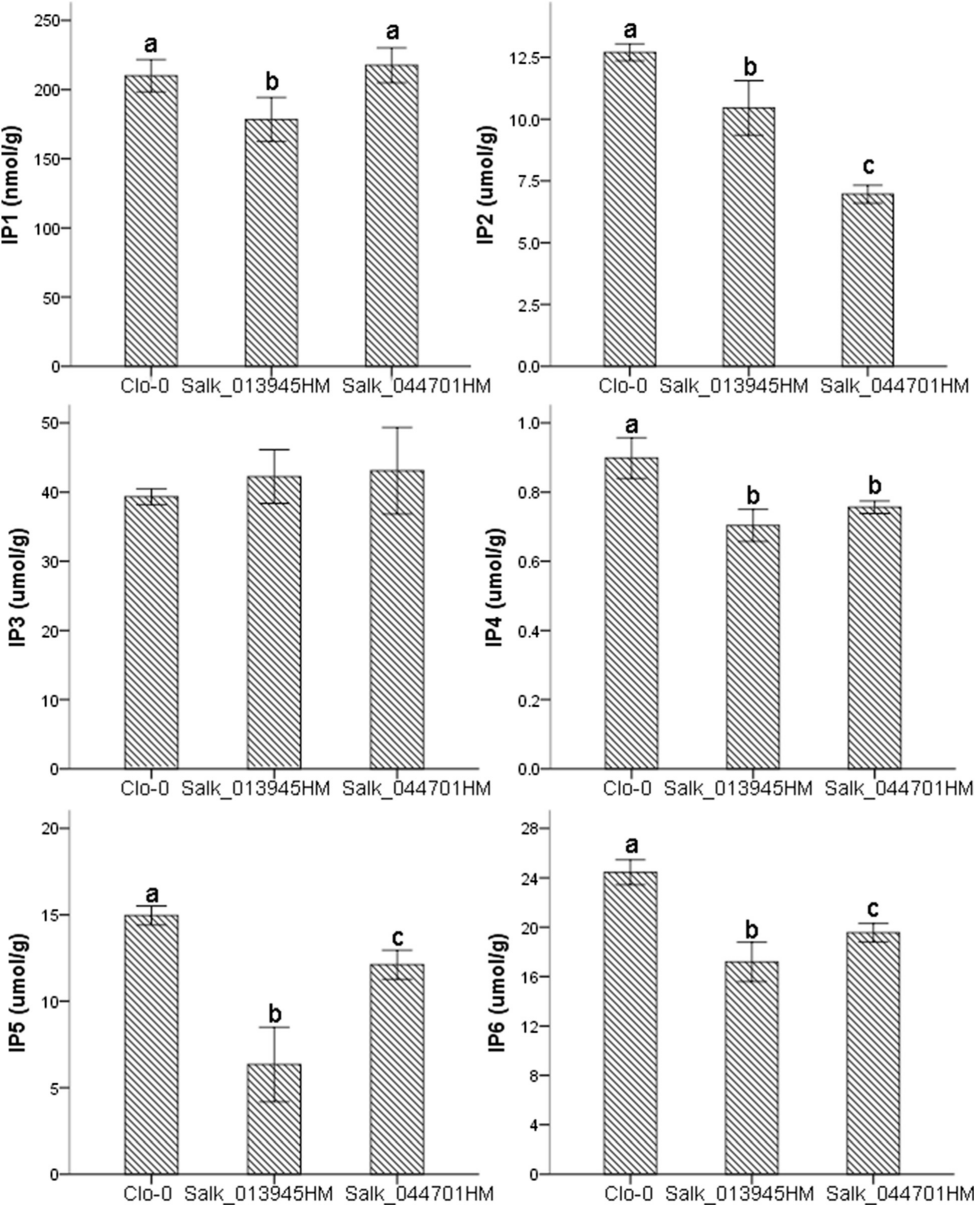
Inositol phosphate levels in seeds of *Arabidopsis* T-DNA insertion lines. Bar represents the standard error. At least three individuals from each insertion line were used for IP determination. Differences in the mean levels of IP in *Clo-0*, Salk_013945 and Salk_044701 were tested using one-way ANOVA. Significant differences were labelled as “a”, “b” and “c” (*p* < 0.05). Lines without significant differences were labelled with the same alphabet or unlabelled. HM: homozygote

To further validate the function of each candidate gene, we obtained T-DNA insertion mutant lines of *Arabidopsis* orthologs of *DH2* (orthologous to AT1G15520; insertion line SALK_013945) and *CB5* (orthologous to AT1G16780; insertion line SALK_044701) (Additional file 1: Figure S17). IP metabolism was found to be perturbed in these two *Arabidopsis* insertion lines, levels of almost all IPs—with the exception of IP3—were decreased in both mutant lines (Fig. 8). The IP6 level was significantly decreased in SALK_013945 (~30 %) and SALK_044701 (~20 %) compared with in *Clo-0*. Interestingly, the IP1 level in SALK_044701 was similar to that in *Clo-0*, but was decreased by ~23 % in SALK_013945. These differences in the IP levels in insertion lines suggest that the *DH2* and *CB5* genes play different roles in IP metabolism. Taken together with the GFP-fused transient transformation data, these results suggest that *DH2* and *CB5* participate in IP transport across organs and co-transport into vesicles, respectively.

**Fig. 9:**
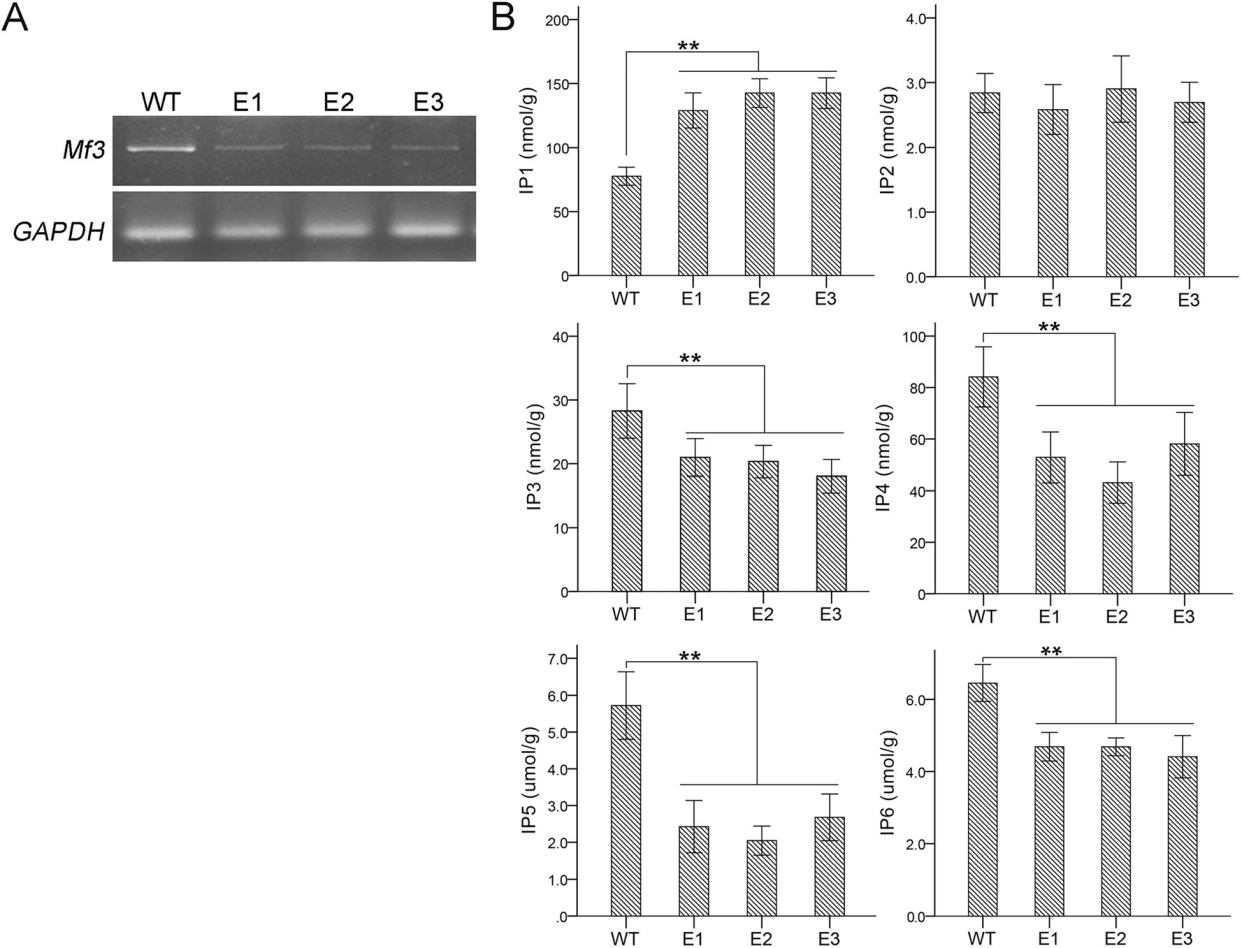
Inositol phosphate levels in maize RNAi lines. **a** RT-PCR of *Mf3* in maize RNAi lines. E1, E2 and E3, different transgenic events/lines; WT, wild-type, negative lines segregated from transgenic lines; *GAPDH*, glyceraldehyde 3-phosphate dehydrogenase gene (used as a reference gene). **b** IP levels in seeds of maize transgenic lines. E1, E2 and E3 represent RNAi transgenic lines. **, significant differences between E1, E2 or E3 and WT (negative transgenic lines) were determined using Student’s t-test (*p <* 0.01)

To validate the function of *Mf3*, we ligated an *Mf3* cDNA segment into pCAMBIA3301 with a ubiquitin (UBI) promoter. The transcript produced from the segment formed a hairpin structure (for RNA interference, RNAi). The construct was transformed into maize to produce *Mf3*-knockdown lines (Fig. 9a, RT-PCR validation of the knockdown levels of *Mf3*), and the IP levels in the knockdown lines were then determined (Fig. 9b). Results showed that, the expression level of *Mf3* in the transgenic lines was reduced approximately three- to five-fold compared with the segregated negative transgenic lines. The IP1 level increased almost two-fold in the RNAi transgenic lines, but the IP2 level showed no significant difference. However, the levels of IP3–IP6 decreased significantly in the RNAi lines. That of IP6 decreased ~30 % compared to the transgenic negative lines. Taken together with the GFP-fused transient transformation results, the findings suggest that the product of the *Mf3* gene functions as an enzyme in IP metabolism.

## Conclusions

To explore the regulatory mechanism of IP metabolism, we screened two inbred lines with significantly different IP6 levels (B73 and Qi319) from a maize germplasm collection and carried out RNA-Seq and microRNA-Seq analyses.

Transcriptome analyses showed that *IPK/ITPK* expression was upregulated at 12DAP in B73 compared with Qi319, while most known genes were downregulated in B73 after 12DAP. However, *MIPS* and *ITPK* showed continuously different expression patterns between the two lines. The differences in gene expression patterns were related to the abundance of IPs (particularly IP4 and IP6) in embryos, suggesting a different pattern of IP metabolism regulation between the B73 and Qi319 inbred lines.

Several transcription factors, especially WRKY and ethylene-responsive transcription factors, would be involved in the regulation of IP metabolism. Moreover, three microRNAs, which would be involved in IP metabolism regulation were identified. These findings will facilitate further research into IP metabolism.

To assess the implications of our data, six co-regulated networks were constructed. These networks have the potential to uncover the function and mechanism of regulation of IP metabolism.

The networks suggest Ca^2+^ as the bridge and core node between IP metabolism and other pathways, and inositol-phosphate-related genes were linked with GA-related genes through ubiquitin-related genes or specific transporter genes (see networks “magenta2” and “dodgerblue4”). This information will facilitate investigation of the interactions of IPs with GA. We also inferred several carbohydrate/inositol transporters and specific ABC transporters as responsible for inositol or IP transport across organs.

Three new candidate genes were extracted from the networks and validated experimentally. Gene mutations in *Arabidopsis* and gene knockdown in maize showed that the candidate genes were indeed involved in IP metabolism. The *Mf3* gene (“magenta2” network) encodes an enzyme, *DH2* (“dodgerblue4” network) encodes an ABC transporter responsible for cross-organ transport of IP, and the product of the *CB5* gene (“cornsilk” network) is related to the co-transport of IP into vesicles.

## Methods

### Plant materials and IP determination

Four hundred seventy-five maize inbred lines (an inbred maize collection) were cultivated in Sanya (Hainan, 2012), Sanya (Hainan, 2013) and Shunyi (Beijing, 2013), and mature seeds were harvested. IP levels were determined by liquid chromatography coupled with tandem mass spectrometry (LC-MS/MS). Significant differences in IP6 content were evaluated by calculating IP6 content ratios as the function Q = *abs[log*_*2*_*(x*_*i*_*/x*_*j*_*)]*, where “abs” is the absolute value of *log*_*2*_*(x*_*i*_*/x*_*j*_*)*, and *x*_*i*_ and *x*_*j*_ are the IP6 contents of each inbred line. Then, a t-test was performed (*p* < 0.05 was considered to indicate an unstable Q value) by using the Q value to screen the inbred line pairs with stable Q value (Q > 0.58) in phytic acid content. Four maize inbred lines with significant differences in phytic acid (IP6) content—B73 (high in phytic acid, HPA), Qi319 (low in phytic acid, LPA), Lv28 (HPA), and CMBIA141 (LPA)—were first monitored for dynamic changes of each IP in the developmental embryo and kernels. Only B73 and Qi319 were used for RNA-Seq analysis because Lv28 and CIMBL141 were not growing well in the field and were susceptible to diseases and insect pests. Seeds and embryos used for IP monitoring and RNA-Seq analysis were collected from the field (Shunyi, Beijing, 2013) at various days after pollination (DAP, 6DAP, 12DAP, 18DAP, 21DAP, 24DAP, and 30DAP). Dynamic changes in IP levels in the fresh samples were determined by LC-MS/MS.

### RNA-Seq and microRNA-Seq

Embryos of B73 and Qi319 (12DAP, 21DAP, and 30DAP) were subjected to RNA-Seq and microRNA-Seq analyses. Total RNA was isolated from embryos using an RNeasy Plant Mini Kit (74904; Qiagen, Germany) according to the manufacturer’s protocol. MicroRNA was extracted using an miRNeasy Mini Kit (217004; Qiagen, Germany).

Approximately 35 μg of total RNA were used for cDNA synthesis and library construction. All libraries were sequenced using the Illumina HiSeq 2500 platform. Small RNAs were first isolated from total RNA using 6 % agarose gels, and then purified. A library was then constructed using the Multiplex Small RNA Library Prep Set for Illumina (NEB).

### Annotation and statistics

Reference maize genome sequences were downloaded from Gramene (B73 AGPv3, http://gramene.org/). Protein sequences were downloaded from Uniprot (http://www.uniprot.org/). ID mapping for DNA and protein sequence matching was accomplished using R (ver. 3.1.2). Statistical analyses were conducted using R. Gene annotation based on BLAST was performed using Blast2GO [77]. The blast E-value was set as 1 × 10^−3^, and the E-Value-Hit-Filter was set as 1 × 10^−6^ in Blast2GO.

### Read alignment and assembly

For RNA-Seq, all reads from each sample were aligned to the reference genome of maize using TopHat2 [78]. Briefly, reads were first aligned to the reference genome by using Bowtie [79] to identify splice junctions between exons and then the aligned reads were subjected to Cufflinks [80] to assemble those reads into sample-specific transcriptomes using alignment coordinates.

For microRNA-Seq, high-quality reads were aligned to the GenBank and Rfam [81] databases to remove ncRNAs, with the exception of miRNAs. miRNAs were identified using miRDeep2 software [82]. Target prediction for known and unknown miRNAs was performed as reported previously using TargetFinder software [83]. Briefly, miRNA sequences were matched to the reference mRNA FASTA sequences and potential targets were computationally predicted by the match/mismatch-scoring ratio. Only predicted targets with scores less than four were considered reasonable.

### Gene/miRNA differential expression analysis

Gene expression levels were computed and expressed as reads per Kb per million fragments (RPKM), which was defined as:

$$ \mathrm{RPKM} = \mathrm{total}\ \mathrm{exon}\ \mathrm{reads}/\left[\mathrm{mapped}\ \mathrm{reads}\ \left(\mathrm{millions}\right)\ \mathrm{exon}\ \mathrm{length}\ \left(\mathrm{K}\mathrm{b}\right)\right]. $$

Differential gene/miRNA expression was analyzed using DESeq [84]. DEGs were identified by calculating the fold-change ratios (FC) between samples. Genes with FC ≥ 2 and FDR < 0.01 were considered differentially expressed and their levels of up- and down-regulation were expressed as logarithms of FC (log_2_FC).

### Alternative splicing analysis

Various types of alternative splicing (AS)—exon skipping (ES), intron retention (IR), mutually exclusive exon (MEE), alternative first exon (AFE), alternative last exon (ALE), alternative 5’ splice site (5’AS) and alternative 3’ splice site (3’AS)—were analyzed using Cufflinks. The novel spliced exons were identified by comparing the sequenced gene model with the annotated locus. The detected novel AS models were visualized using SpliceGrapher [85], giving a diagram view.

### Novel transcripts analysis

All reads from RNA-Seq were first assembled using TopHat2 and Cufflinks. Exons and junctions that overlapped or were adjacent to the existing annotated transcripts were filtered out. Finally, the remaining exons were extended and merged. The novel assembled transcripts were future filtered according to their size (coding sequence > 150 amino acids). The novel transcripts were annotated using Blast2GO.

### Network construction and analysis

RNA-Seq gene expression data were filtered such that genes with more than three missing values or 0 were filtered out. Ultimately, 15,536 genes were filtered out and 26,264 genes were retained for further processing.

Gene correlation coefficients (Spearman’s coefficient, ρ) were calculated using the WGCNA R package [86], but the maximum block size was set as 3500 to save running time, and the threshold for network output was set as 0.5 to achieve more stringent connectivity of nodes in the network. Co-expressed genes were clustered by applying ***TOM*** and ***DynamicTreeCut*** functions to form different co-expression modules. A unique color was assembled to name each module. Correlations of IPs with modules were calculated using the ***cor()*** function in R and the significance of the correlations was evaluated by t*-*tests. Correlation coefficients > 0.80 and *p*-values < 0.01 were considered to indicate a significant correlation.

Modules that were significantly correlated with IPs were extracted and exported to Cytoscape (ver. 2.8, http://www.cytoscape.org/) for network visualization and editing [87, 88]. Hub genes were identified using the *cyotHubba* plugin for Cytoscape [89]. Network compression was conducted by the Power Graph method [44, 90, 91] and the networks were compressed according to the linkage of nodes.

To understand the implications of the gene co-expression network, we carried out a literature search using the “AgilentLiteratureSearchPlugin” (ver. 2.78, http://apps.cytoscape.org/apps/) and MiMI Plugin [92] for Cytoscape. Nodes and studies were sorted using the key words “inositol”, “phytic acid”, “phytate”, and “phosphatidylinositol.” Functional elements were extracted according to the GO annotation and literature containing the above-mentioned key words.

Key nodes and candidate genes were selected according to guide-genes found to be involved in IP metabolism in maize. The guide-genes were: *MIK* (GRMZM2G361593) [29], *ITPK* (GRMZM2G456626) [17], *MIPS* (GRMZM2G155242) [93], and *ABC transporter* (GRMZM5G820122) [19].

We used the following principles to screen candidate genes: first, candidate genes and inositol phosphate-related genes should share the same key node/hub gene; secondly, the distance from guide-gene/inositol phosphate-related gene to the candidate gene should be less than 4 [94]; and third, the co-regulated genes that correlated with the guide-genes and candidate genes should be in power nodes. It was considered optimal if conserved domains present in known proteins—including carbohydrate kinase PfkB, IPR011611; P-loop-containing, IPR005337; and P-loop containing nucleoside triphosphate hydrolase, IPR027417 domains—were also present in the proteins encoded by candidate genes. Genes differentially expressed in the B73 and Qi319 lines were also considered.

### Quantitative RT-PCR

Total RNA was extracted with TRIzol reagent (TaKaRa, Dalian, China) according to the manufacturer’s instructions. Total RNA (100 ng) was used for first-strand cDNA synthesis using EasyScript One-Step gDNA Removal and cDNA Synthesis SuperMix (TRANSGEN, Beijing China). Quantitative reverse transcription-polymerase chain reaction (*q*RT-PCR) was performed using TransStart Green qPCR SuperMix (TRANSGEN, Beijing, China). Primers used for *q*RT-PCR are listed in Additional file 7.

### Validation of candidate genes

For convenience, candidate genes were named using the node name of the containing module.

GFP fusion expression was carried out to assess the subcellular localization of each candidate gene in the maize protoplast. Protoplasts of maize B73 were prepared according to Yoo et al*.* [95] using etiolated seedlings, but the time required for protoplast isolation was reduced to 3 h, and ~30 μg of plasmid DNA was used for transient transformation. CDS of candidate genes were ligated into pRTL2 to form a C-fusion GFP construction using restriction enzymes (Additional file 7).

Due to the limited maize mutant resources available, we obtained *Arabidopsis thaliana* lines containing T-DNA insertions in genes orthologous to the candidate genes (Additional file 1: Figure S17) from ABRC (https://abrc.osu.edu/). IP levels in seeds of the insertion lines were determined by LC-MS/MS (at least three individuals of each insertion line were used for IP determination). Gene names and the corresponding *Arabidopsis* mutant lines are listed in Table 3. Mutant test primers are listed in Additional file 7. The T-border primer was LBa1: 5'-TGGTTCACGTAGTGGGCCATCG-3'. T-DNA insertion effects were tested by RT-PCR using cDNAs of siliques at 20 days after flowering. Primers used for RT-PCR are listed in Additional file 7.

The maize *Mf3* gene was evaluated by RNAi technology. The conserved sequence of *Mf3* was reversed and forward-linked to a rice intron to form a hairpin structure and then digested with *Hind*III and *Sac*I from pTCK303 and linked into pCAMBIA3301, which had been digested with *Hind*III and *Bst*EII. The 35S promoter and the GUS coding sequence were then replaced by the *Mf3* RNAi segment (Additional file 1: Figure S18). Primers for *Mf3* segment cloning are listed in Additional file 7. The final expression plasmid vector was transformed into GV3103 (*Agrobacterium tumefaciens*) and then transfected into an immature embryo of HiII, induced into callus, regenerated seedling under selection stress of glufosinate.

In total, five transgenic events were obtained, three of which (based on RT-PCR results in transgenic lines) were used for IP determination. T1 seeds (5–10) of each transgenic line were ground singly using a Geno/Grinder 2010 (Molecular Devices, Sunnyvale, CA USA). A portion of the powder (30 mg) was used for IP determination and the remainder for PCR screening of positive transgenic individuals. *Mf3* knockdown levels were validated by RT-PCR in T1 seedlings of RNAi lines.

### Availability of supporting data

RNA-Seq data used in this study have been deposited into the NCBI Sequence Read Archive (SRA, http://www.ncbi.nlm.nih.gov/sra/) under accession number SRP065818 (SRR2907733, SRR2908040, SRR2908041, SRR2908042, SRR2908043, and SRR2908044).

## Additional files

Additional file 1:
**Figure S1.** to S18. (PDF 3864 kb) Additional file 2:
**Table S1.** Table S2. (DOCX 15 kb) Additional file 3:
**Differentially expressed transcription factors.** (XLSX 20 kb) Additional file 4:
**Sub-network 1 and 2 of “magenta2”, node annotation.** (XLSX 10 kb) Additional file 5:
**“cornsilk” network and node annotation.** (XLSX 9 kb) Additional file 6:
**Sub-network 1 and 2 of “dodgerblue4” network, node annotation.** (XLSX 9 kb) Additional file 7:
**Primers used for cloning, PCR, RT-PCR and** ***q*** **RT-PCR.** (DOCX 15 kb)

## Abbreviations

ABC: ATP-binding cassette
AS: alternative splicing
DAP: days after pollination
DEG: differentially expressed gene
GA: gibberellic acid
HPA: high phytic acid content
IP(s): *D*-myo-inositol phosphate(s)
ITPK: inositol-trisphosphate 3-kinase
LC-MS/MS: liquid chromatography coupled with tandem mass spectrometry
LPA: low phytic acid content
lpa: low phytic acid
MIK: myo-inositol kinase
MIPS: myo-inositol 1-phosphate synthase
MRP: multidrug resistance-associated protein
ncRNA: non-coding RNA
PLC: phospholipase C
TFs: transcription factors
WGCNA: weighted gene co-expression network analysis
