# Supplementary material for: Analysis of weighted co-regulatory networks in maize provides insights into new genes and regulatory mechanisms related to inositol phosphate metabolism
Source: BMC Genomics. 2016 Feb 24;17:129. doi: 10.1186/s12864-016-2476-x (PMC4765147; doi:10.1186/s12864-016-2476-x)
Supplement: Additional file 1: — Figure S1. to S18. (PDF 3864 kb) [file 12864_2016_2476_MOESM1_ESM.pdf]

Figure S1-S18

Figure S1

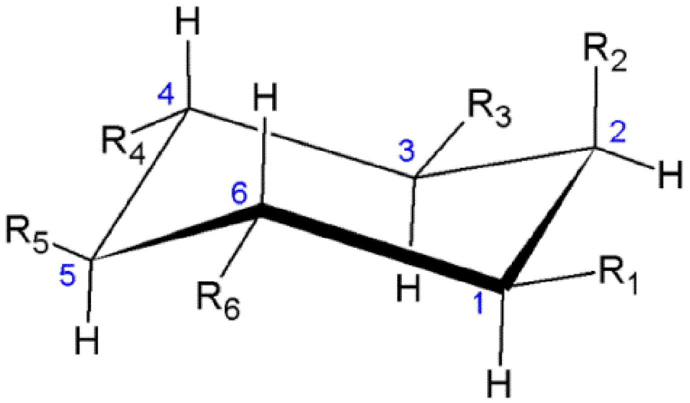

Figure S1 Molecular structures of *D-myo*-inositol phosphates. $R_{1\sim6}$ = OH (m) or $OPO(OH)_2$ (n). Inositol: n = 0, m = 6; $InsP_1$: n = 1, m = 5; $InsP_2$: n = 2, m = 4; $InsP_3$: n = 3, m = 3; $InsP_4$: n = 4, m = 2; $InsP_5$: n = 5, m = 1; $InsP_6$: n = 6, m = 0.

Figure S2

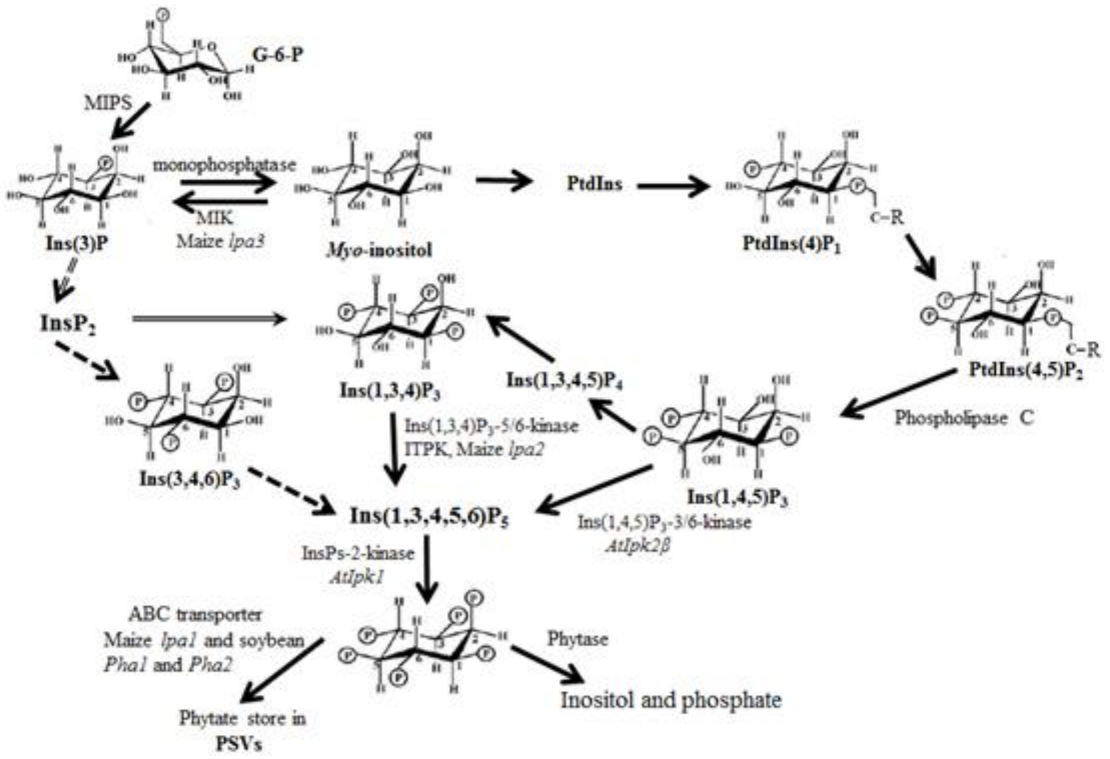

Figure S2 Phytic acid synthesis pathways in plants. The molecules draw in diagram represent the more abundance structures of each inositol phosphate in plants. The normal font on the arrows are enzymes involved in phytic acid synthesis, low phytic acid mutant lines (*lpa*) are emphasized in italics. The dash lines mean no genes or enzymes are discovered at present. Lift: lipid-independent pathway. Right: lipid-dependent pathway, from PtIns to Ins(1,4,5)P_3; PtIns, phosphatidylinositol.

Figure S3

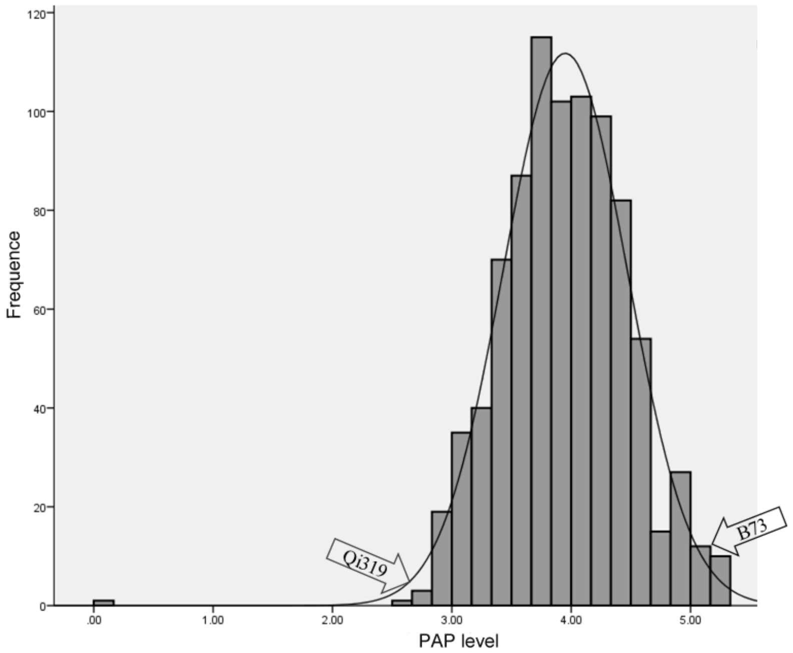

Figure S3 Frequency histogram of phytic acid phosphor (PAP) levels in 425 maize inbred lines. Content data were collected from three points (in two years and at two different locations) and the best linear unbiased prediction (BLUP) was performed in R, the BLUP results were used for this frequency histogram. Arrows indicate the PAP content ranges of B73 (high-in-phytic-acid) and Qi319 (low-in-phytic-acid).

Figure S4

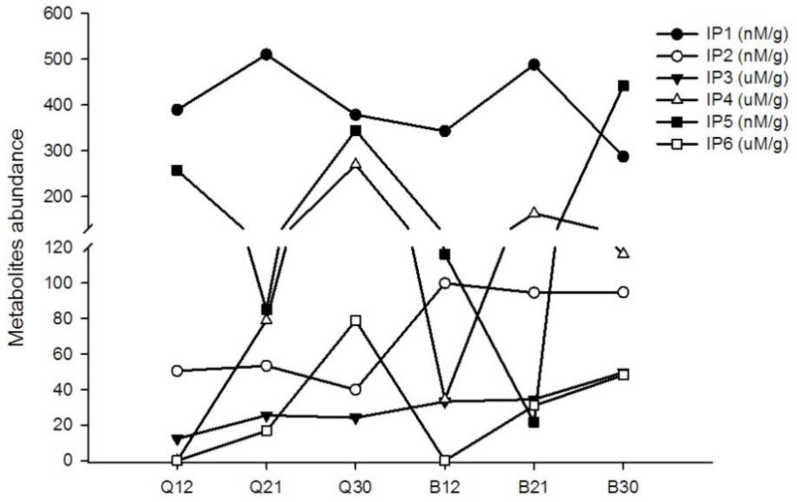

Figure S4 IP1-IP6 levels in fresh embryo of B73 and Qi319. B12, B21, B30, B73 embryo at 12, 21 and 30DAP respectively. Q12, Q21, Q30, Qi319 embryo at 12, 21 and 30DAP.

Figure S5

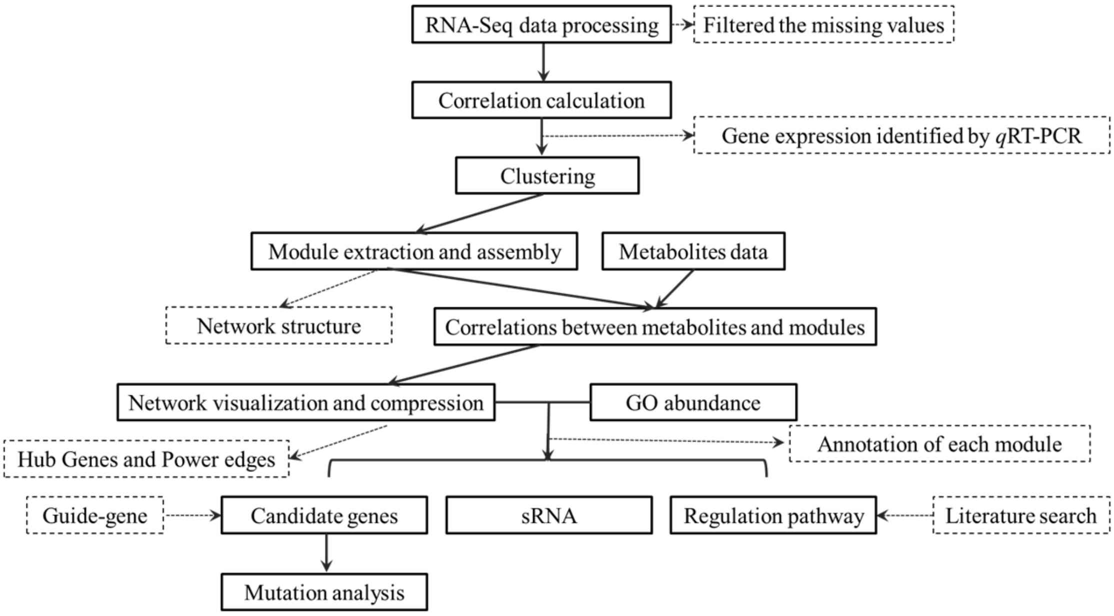

Figure S5 Workflow for data processing and analysis (see also materials and methods)**.**

Figure S6

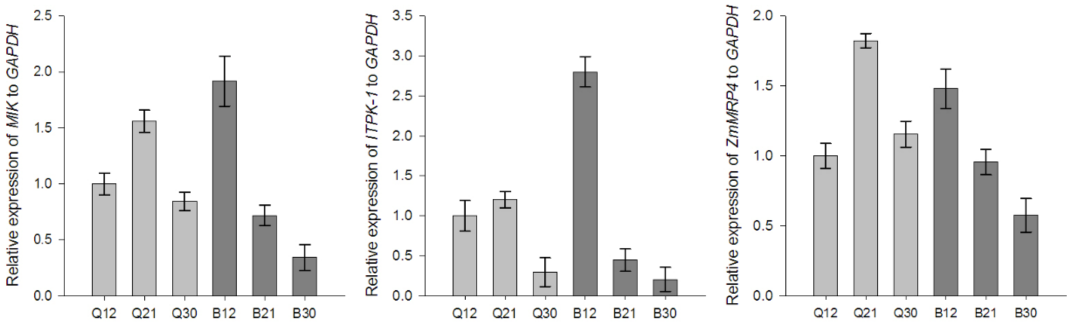

Figure S6 The relative expression levels of the three known genes in maize embryo. Q12, Q21, Q30: embryo at 12, 21, 30DAP of Qi319, grey histogram; B12, B21, B30: embryo at 12, 21, 30DAP of B73, dark grey histogram. The relative expression levels of each gene were evaluated by *q*RT-PCR (ΔΔCt), error bars indicate the standard deviations of three replicates. *MIK*, *myo*-inositol kinase gene (*lpa3*), GRMZM2G361593. *ITPK-1*: inositol-1,3,4-trisphosphate 5/6-kinase gene (*lpa2*), GRMZM2G456626. *ZmMRP4*, ABC transporter gene (*lpa1*), GRMZM5G820122. *GAPDH*: glyceraldehyde 3-phosphate dehydrogenase gene, used as reference gene.

Figure S7

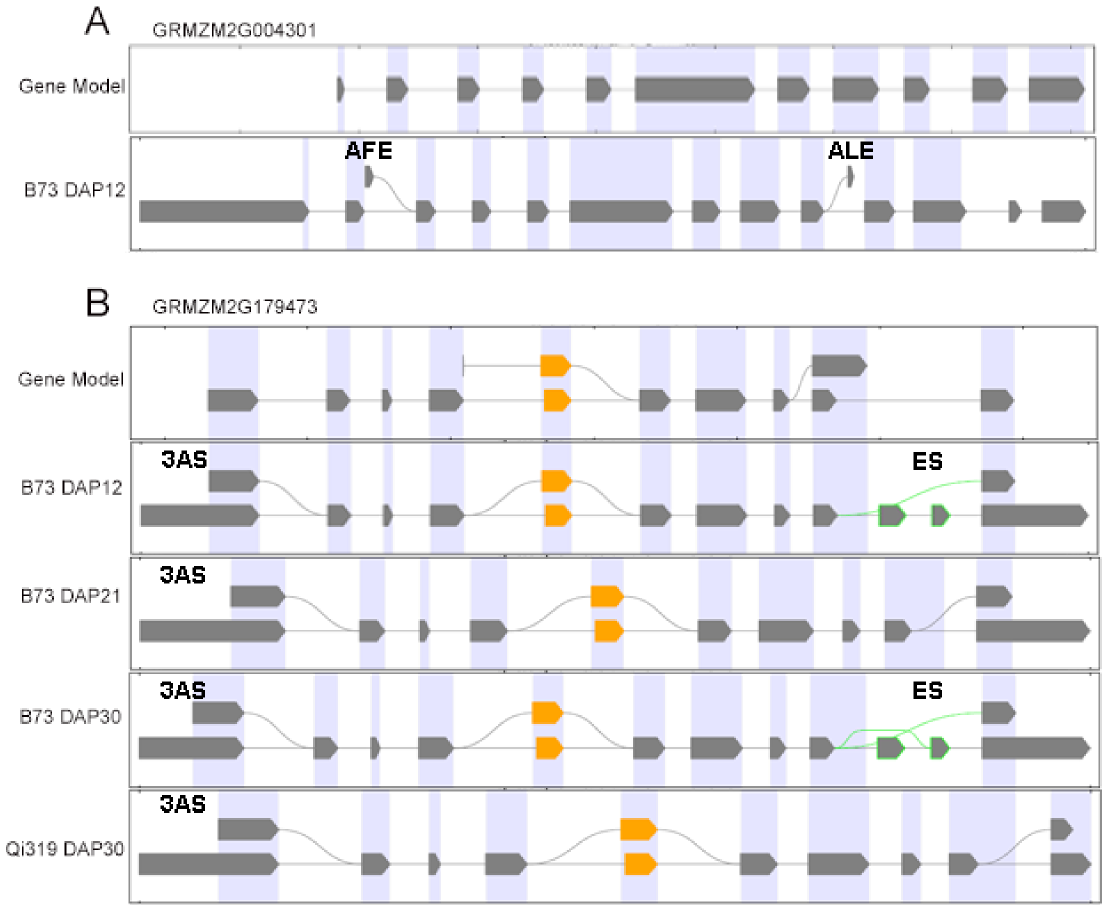

Figure S7 Alternative splicing models of inositol 1,4,5-triphosphate 5-phosphatase gene (GRMZM2G004301, A) and *ITPK-2* (GRMZM2G179473, B) in B73 and Qi319. DAP, days after pollination. Gene Model, the annotated gene model in maize genome database (B73 AGPv3). The following diagram are novel alternative splicing models implied by RNA-Seq. AFE, alternative first exon. ALE: alternative last exon. 3AS, alternative 3' splice site. ES, exon skipping.

Figure S8

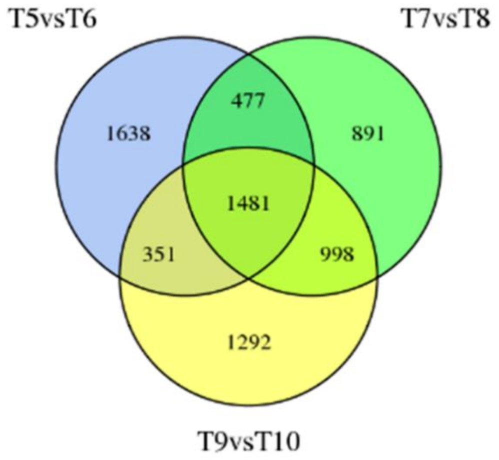

Figure S8 Statistics of differentially expressed genes. T5, T7, T9: transcriptome at 12, 21, 30DAP of Qi319. T6, T8, T10: transcriptome at 12, 21, 30DAP of B73.

Figure S9

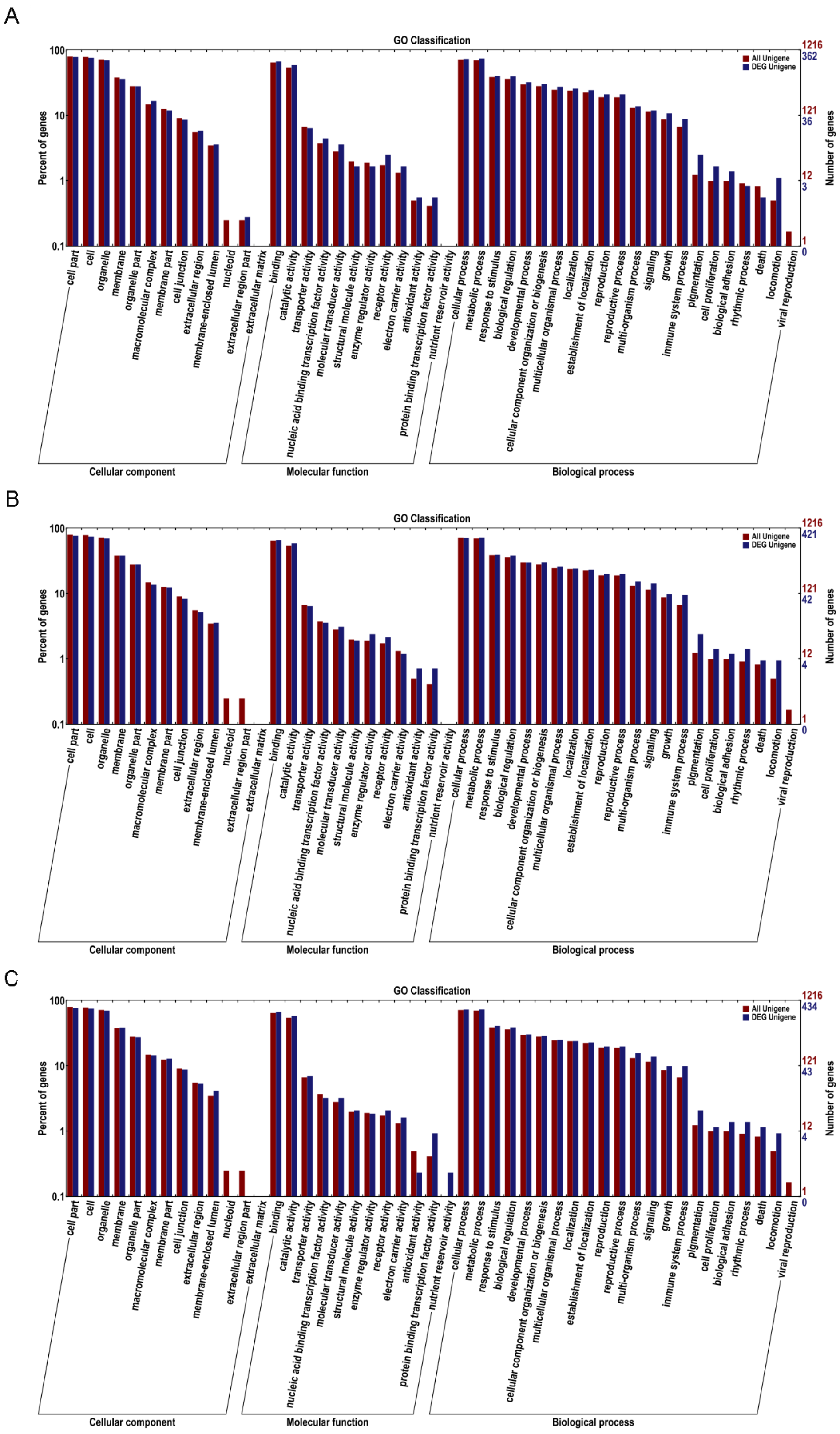

(*Continued on Next Page*)

(Figure S9 continued)

D

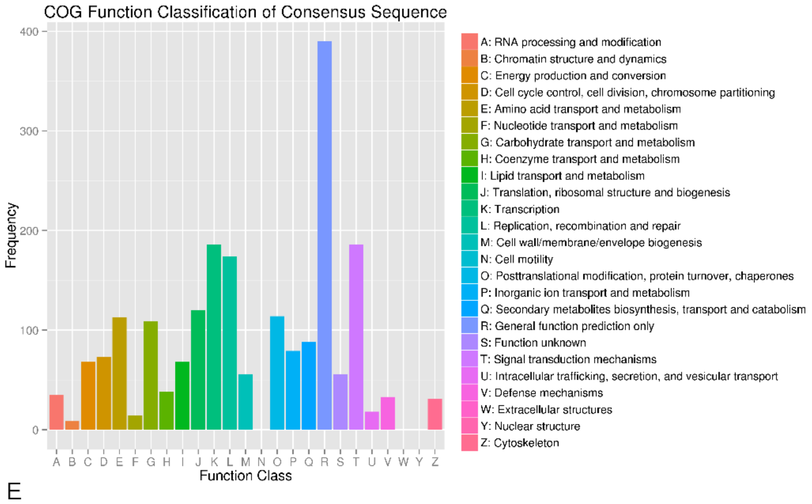

E

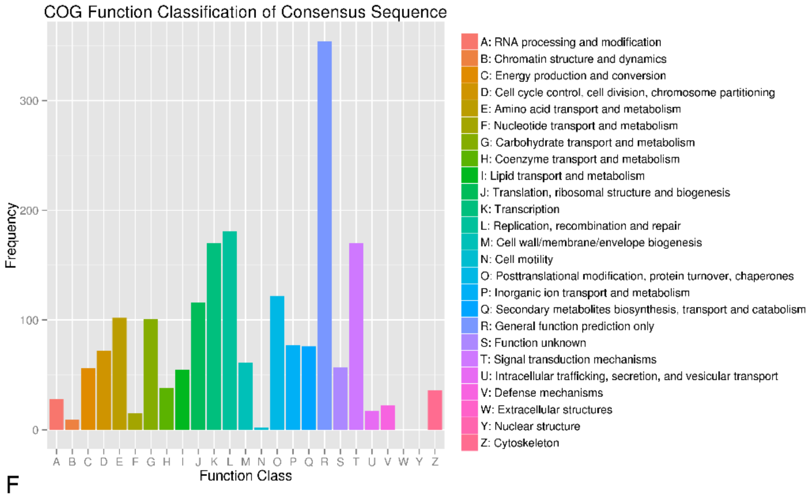

F

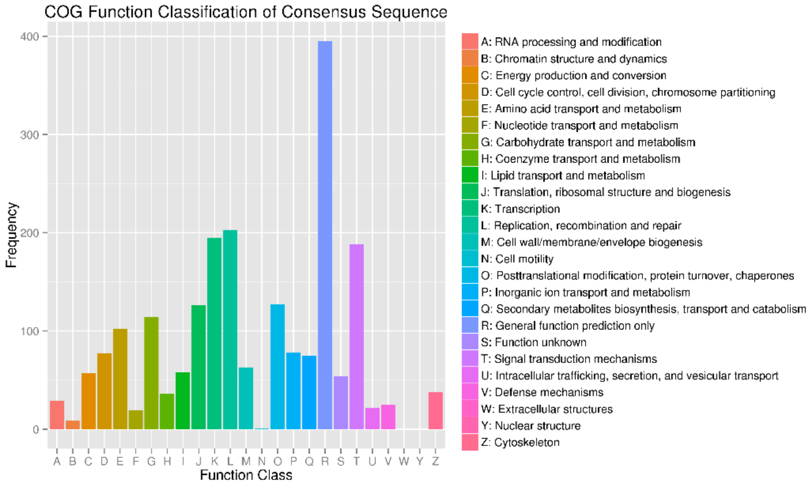

Figure S9 GO and COG annotations of differentially expressed genes. A-C: GO annotation of differentially expressed genes at 12DAP, 21DAP and 30DAP. D-F: COG annotation of differentially expressed genes at 12DAP, 21DAP and 30DAP.

Figure S10

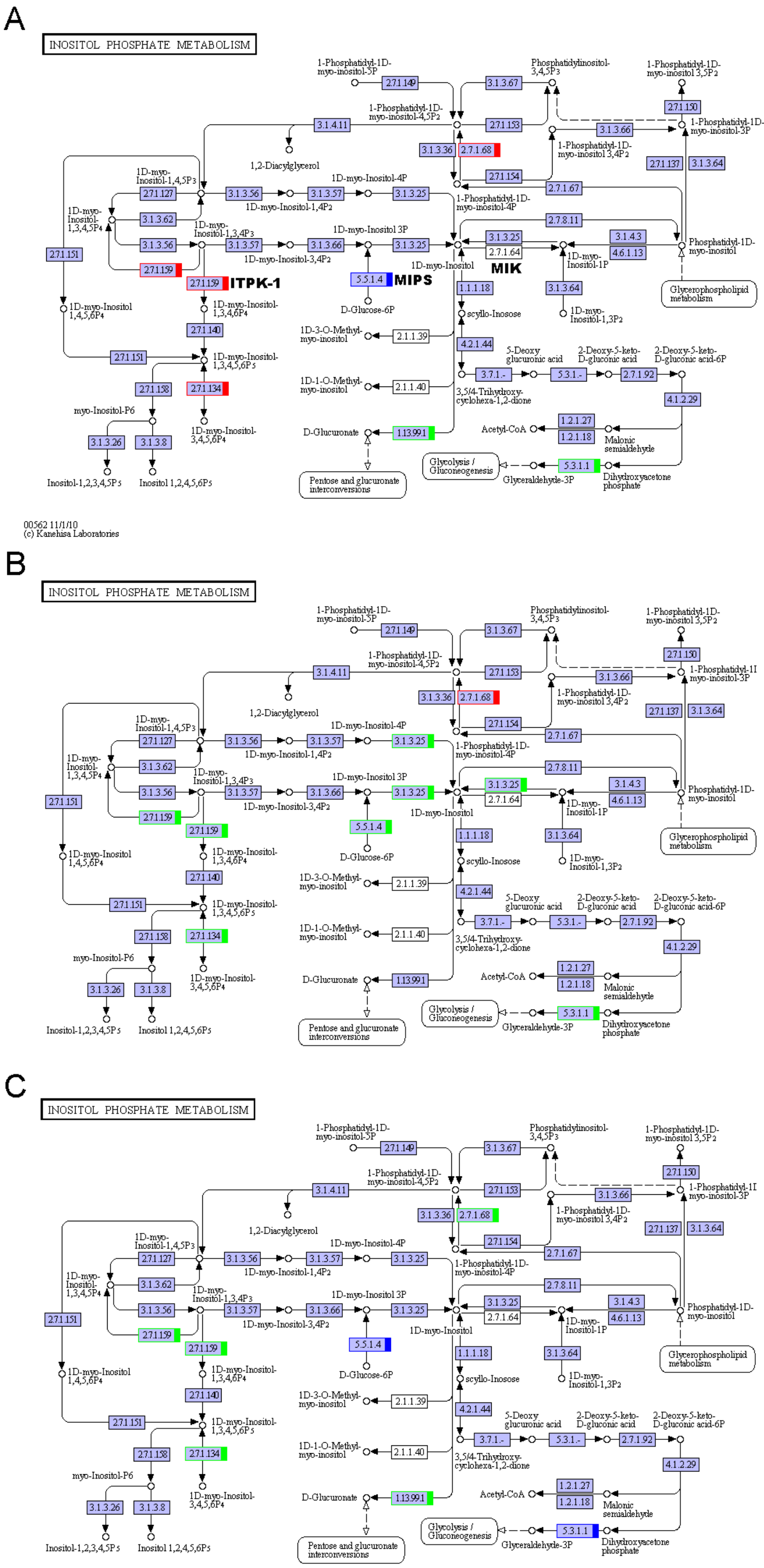

(*Continued on Next Page*)

(Figure S10 continued)

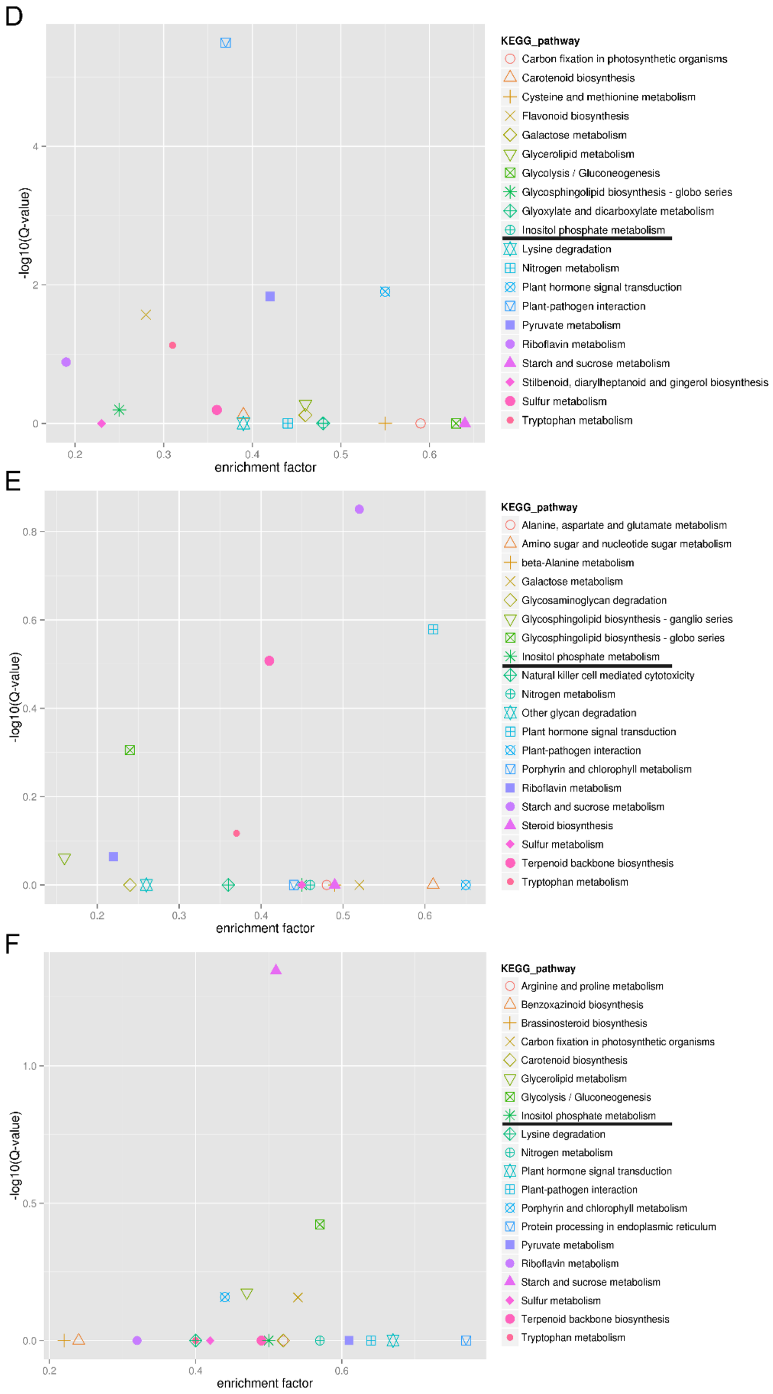

Figure S10 Differentially expressed genes at 12DAP (A), 21DAP (B) and 30DAP (C) were mapped to the KEGG pathway (map00562, inositol phosphate metabolism)**.** Red, genes upregulated in B73; green, genes downregulated in B73; blue, genes up- or down-regulated. Maize enzymes are indicated by bold font. MIK: myo-inositol kinase, *lpa1* of maize; ITPK-1: inositol-1,3,4-trisphosphate 5/6-kinase, *lpa2* of maize; MIPS: inositol-3-phosphate synthase. D-F: gene enrichment in KEGG pathways. The "enrichment factor" represents the percentage of the differentially expressed genes versus all the genes mapped to the same pathway. Q-value was the *p*-value derived from multiple hypothesis testing.

Figure S11

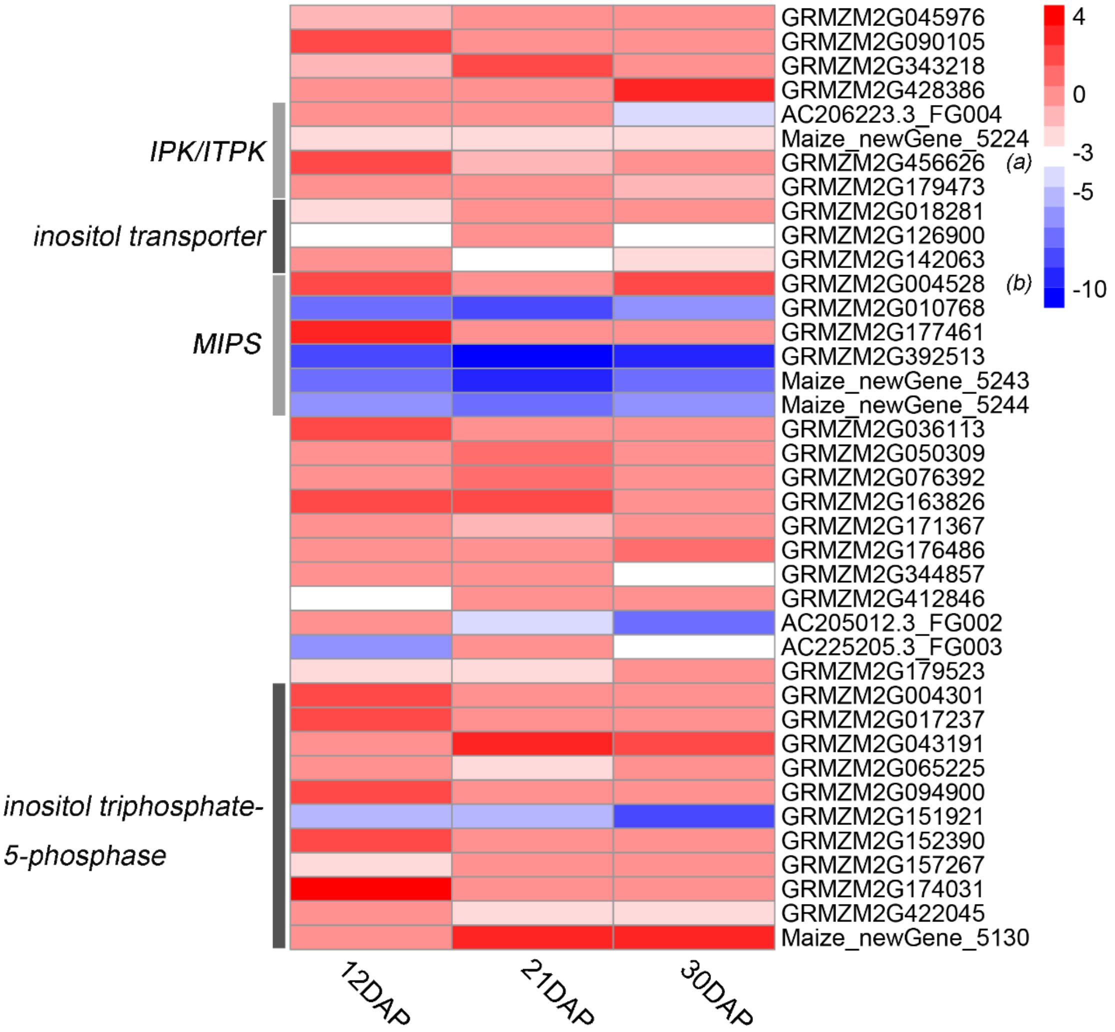

Figure S11 Changes in the expression levels of DEGs with annotation related to inositol phosphate at different developmental stages of embryo. (a), *ITPK-1*, *lpa2* mutant of maize; (b), *MIPS2* gene of maize. DAP: days after pollination. The red color in heat map represents gene up-regulated in B73 and the blue color represents gene down-regulated in B73.

Figure S12

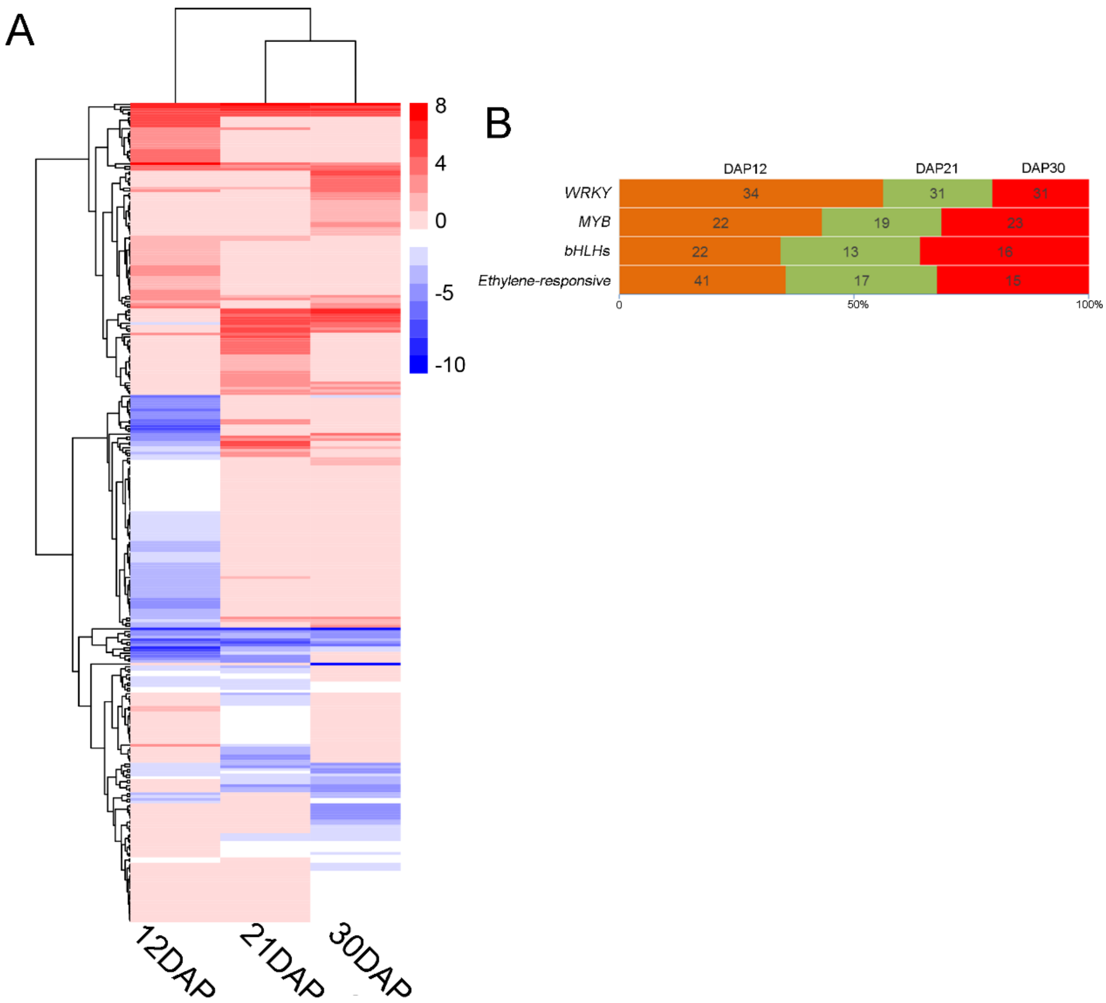

Figure S12 Differentially expressed transcription factors. A, Expression change degrees of differentially expressed transcription factors. Colors represent the natural logarithm values of the expression fold change. DAP: days after pollination. B, The differentially expressed TFs enriched in four TF families. The values in the box are numbers of differentially expressed TFs. DAP: days after pollination.

Figure S13

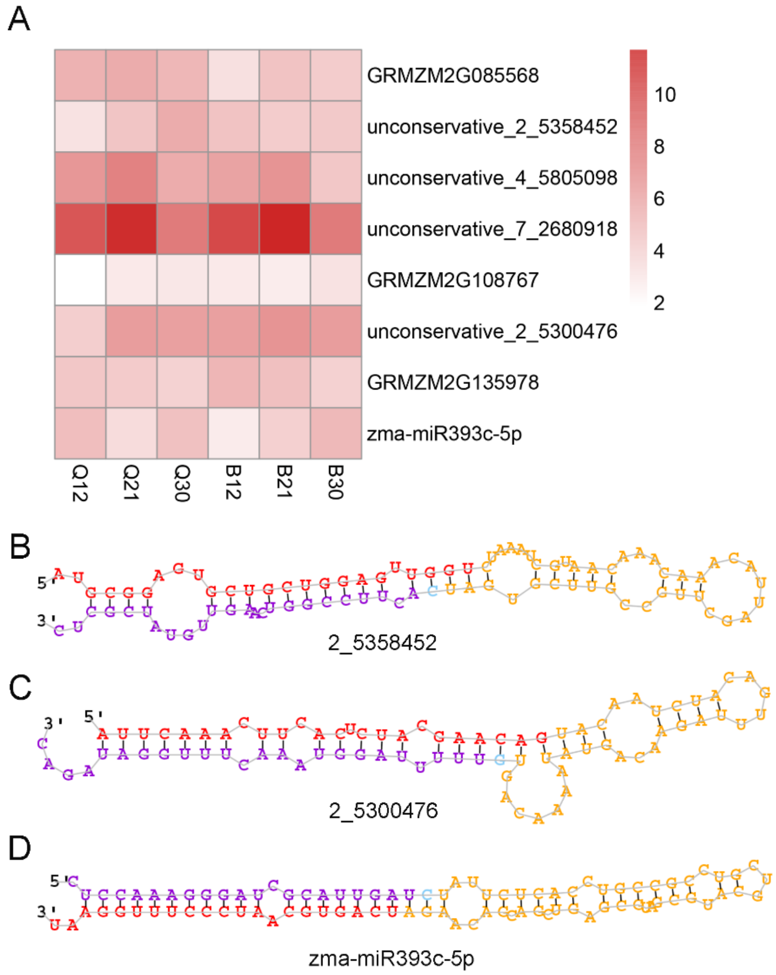

Figure S13 Structures and expression of candidate microRNAs. A, expression patterns of microRNAs and their predicted target genes; the expression level was extracted from the sequencing data, using the binary logarithm value of RPKM. B-D, predicted structures of the selected three microRNAs; the three microRNA were select for structure prediction based on their expression patterns versus their target genes. Red characters were the mature sequence of microRNA.

Figure S14

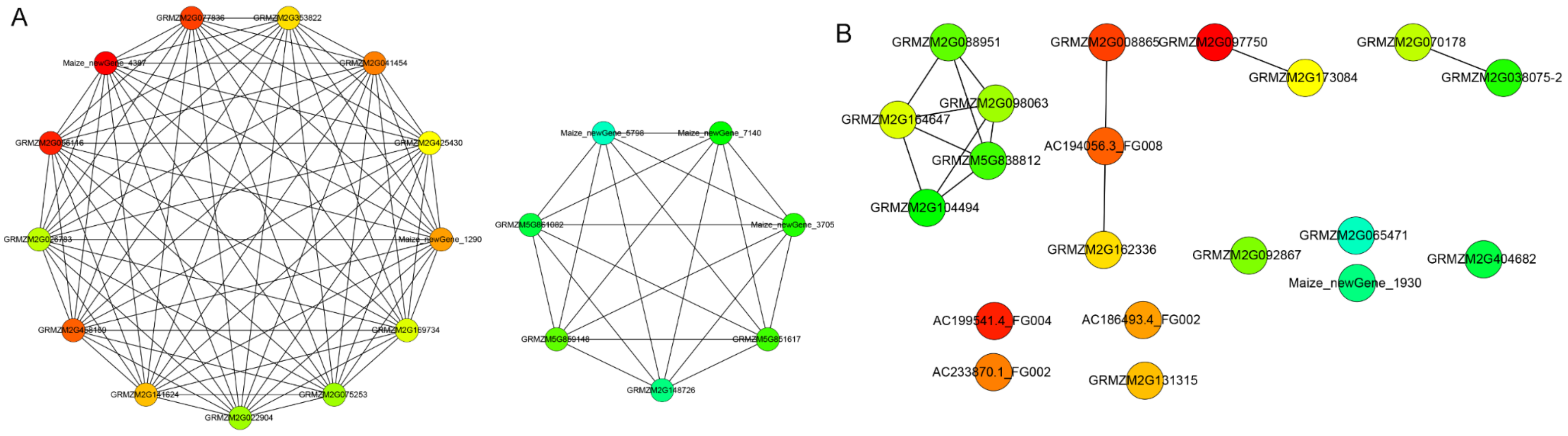

(***Continued on Next Page***)

(Figure S14 continued)

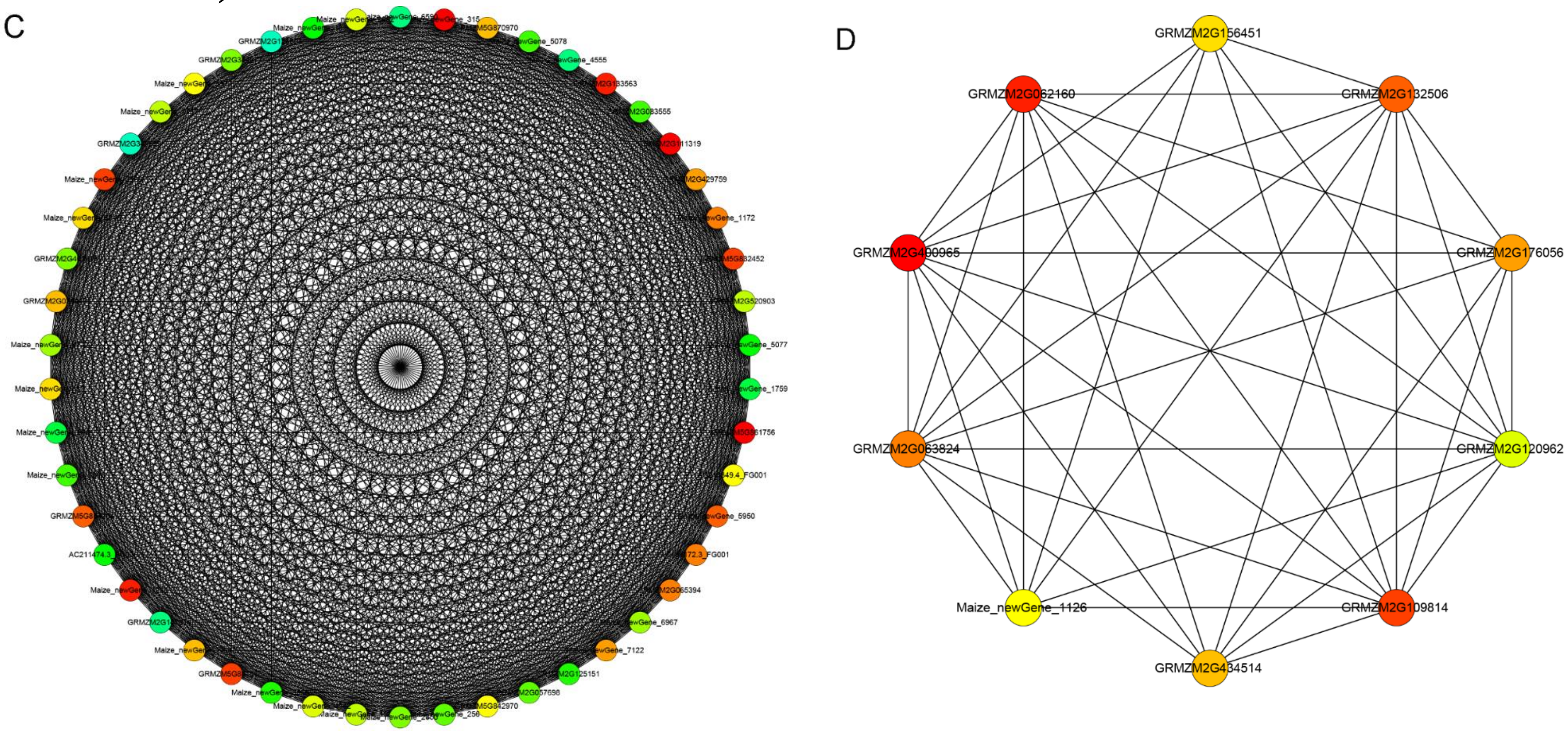

(*Continued on Next Page*)

(Figure S14 continued)

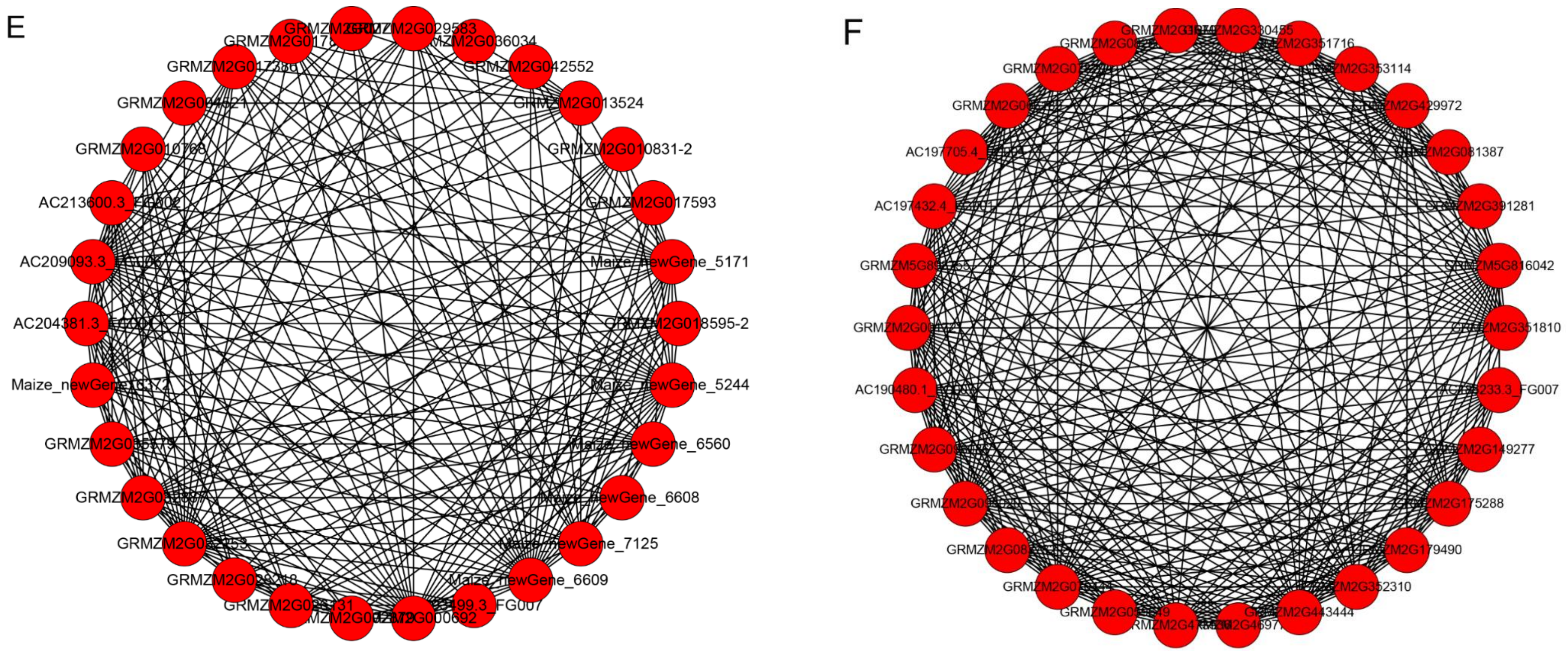

Figure S14 Top 50 hub-genes of the inositol phosphate related gene co-expression networks (see also Table 2)**.** A, “dodgerblue4”. B, “salmon1”. C, “burlywood2”. D, “magenta2”. E, “cornsilk”. F, “steelblue4”. Accession numbers (MaizeGDB) were given on the nodes. Modules “magenta2”, “cornsilk” and “dodgerblue4” were selected for further analysis.

Figure S15

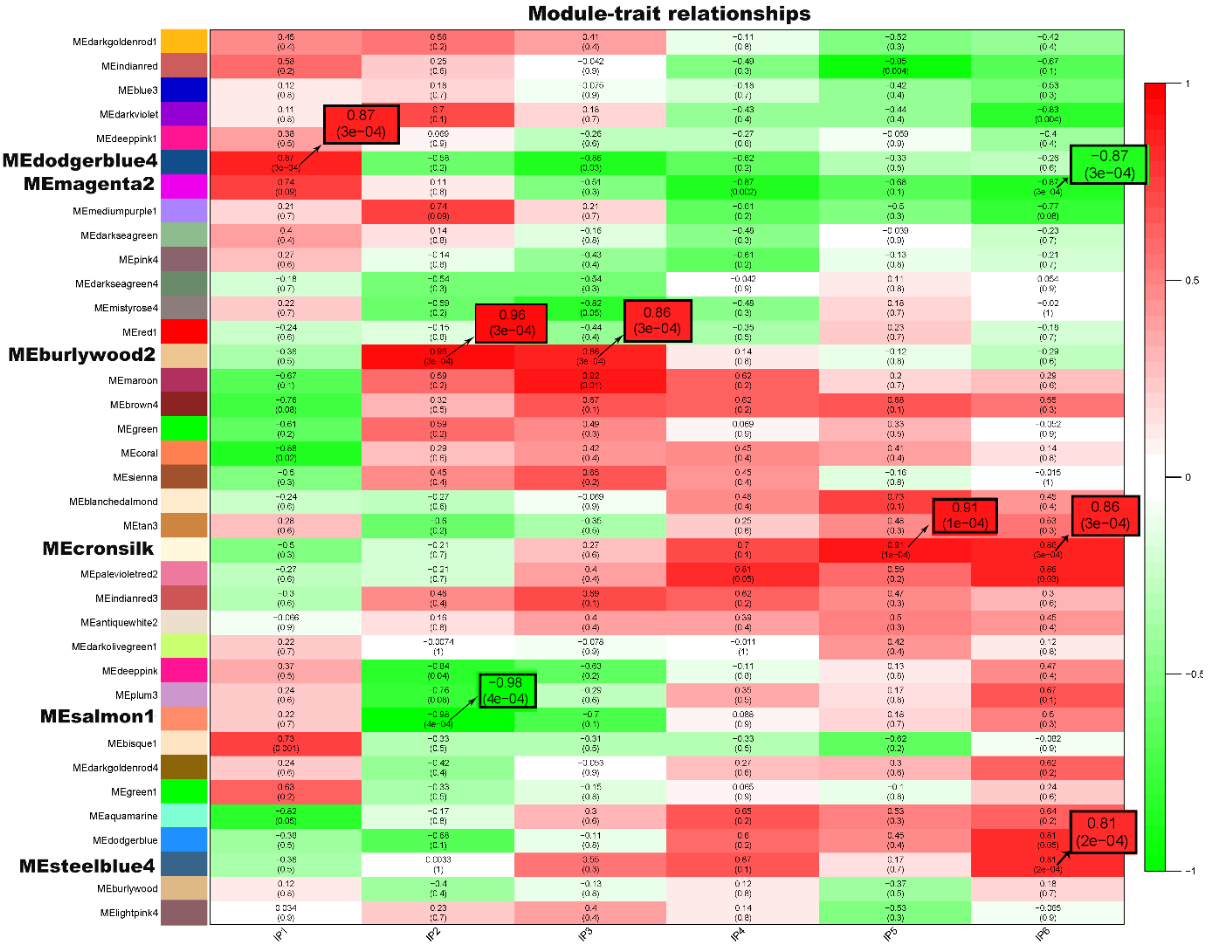

Figure S15 Gene co-expression module and trait relationships. Bold font (on the left) are the six IP-related gene modules with $p < 0.01$ (*t-test*). The upper value in each cell is the correlation coefficient, the lower in bracket is the p value. Modules "indianred" and "darkviolet" were excluded because we could not find guide genes based on node annotation. Modules "magenta2", "cornsilk" and "dodgerblue4" were selected for further analysis.

Figure S16

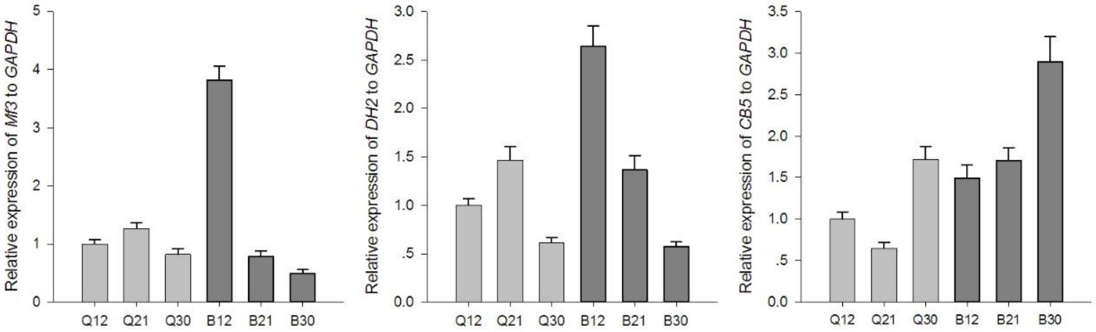

Figure S16 The relative expression levels of the three candidate genes (*Mf3*, *DH2*, *CB5*) in maize embryo. Q12, Q21, Q30: embryo at 12, 21, 30DAP of Qi319; B12, B21, B30: embryo at 12, 21, 30DAP of B73. The relative expression levels of each gene were evaluated by *q*RT-PCR (ΔΔCt), error bars indicate the standard deviations of three replicates.

Figure S17

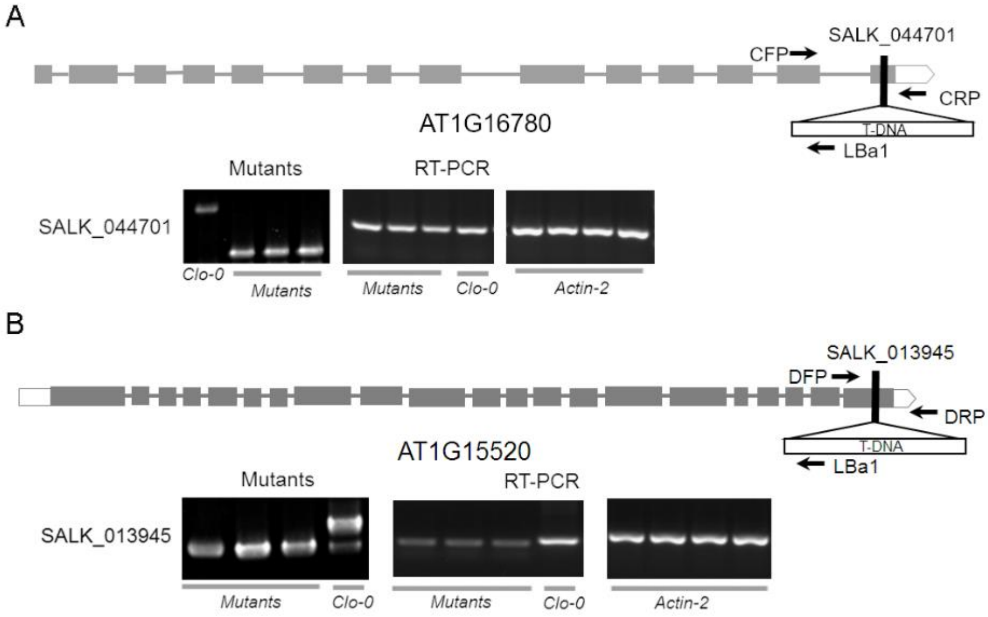

Figure S17 T-DNA insertion lines of *Arabidopsis* orthologs of *CB5* and *DH2*. AT1G16780 is homologous to candidate gene *CB5* (A) and AT1G15520 to candidate gene *DH2* (B) (see also Table 3). Arrows are primers used for mutant screening. On gene model diagram: grey rectangles represent exons, and lines indicate introns. Vertical line indicates the T-DNA inserted position in *Arabidopsis* gene. Mutants: PCR screening of mutant lines. RT-PCR were performed to evaluate the T-DNA insertion effects in mutant lines and *Clo-0* (wild-type). *Actin-2*, reference gene.

Figure S18

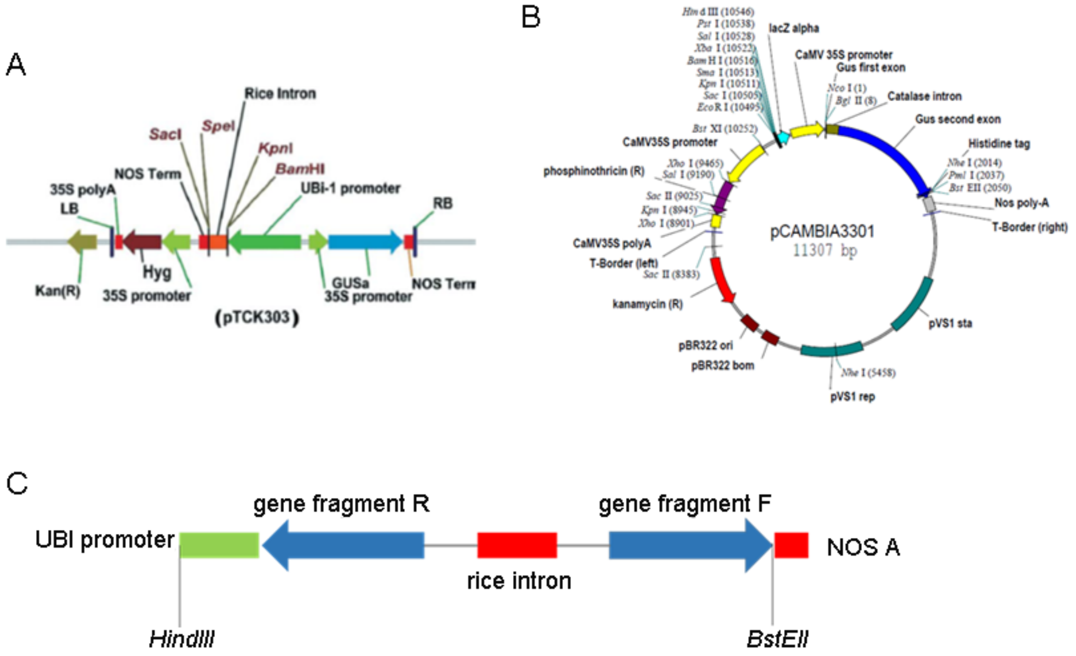

Figure S18 Plasmid vectors used for RNAi construction of gene *Mf3*. A, restriction enzymes map of pTCK303. B, restriction enzymes map of pCAMBIA3301. C, functional structure of *Mf3* RNAi segment: gene fragment R complement with gene fragment F, forming a hairpin structure with rice intron under the control of UBI promoter; 35S promoter and GUS coding sequence in pCAMBIA3301 were replaced by this segment. Primers used for vector construction see Additional file 7. The detailed information see Materials and Methods.
