# Supplementary material for: Analysis of weighted co-regulatory networks in maize provides insights into new genes and regulatory mechanisms related to inositol phosphate metabolism
Source: BMC Genomics. 2016 Feb 24;17:129. doi: 10.1186/s12864-016-2476-x (PMC4765147; doi:10.1186/s12864-016-2476-x)
Supplement: Additional file 2: — Table S1. Table S2. (DOCX 15 kb) [file 12864_2016_2476_MOESM2_ESM.docx]

**Table S1-S2**

**Table S1** The first 24 pairs with significant difference of PAP content ^a^

| Lines | B73 | Lv28 | 1323 | Dong237 | 04K5686 | M153 |
| --- | --- | --- | --- | --- | --- | --- |
| Qi319 | 2.1 | 2.2 | 2.0 | 2.3 | 2.2 | 2.1 |
| CIMBL141 | 2.0 | 2.1 | 1.9 | 2.2 | 2.1 | 1.9 |
| TY6 | 1.8 | 1.9 | 1.7 | 2.0 | 1.9 | 1.8 |
| Yu87-1 | 2.0 | 2.1 | 1.9 | 2.2 | 2.0 | 1.9 |

^a^ The values in table are the IP6 content ratios (≥ 1.5 or ≤ 0.67) of inbred lines. Those line-pairs were selected by calculating the “Q” value (Materials and Methods).

**Table S2** Network attributes and compression ratios

| Module name | Nodes | Edges | Average connectivity | Power Graph Edges | Compression |
| --- | --- | --- | --- | --- | --- |
| dodgerblue4 | 1592 | 10737 | 6.7 | 2196 | 80% |
| magenta2 | 3510 | 9951 | 2.8 | 769 | 92% |
| burlywood2 | 1407 | 122587 | 87.1 | 29493 | 76% |
| cronsilk | 304 | 1650 | 5.4 | 257 | 84% |
| salmon1 | 1638 | 31286 | 19.1 | 7724 | 75% |
| steelblue4 | 2016 | 16740 | 8.3 | 2961 | 82% |
