# Supplementary material for: Analysis of weighted co-regulatory networks in maize provides insights into new genes and regulatory mechanisms related to inositol phosphate metabolism
Source: BMC Genomics. 2016 Feb 24;17:129. doi: 10.1186/s12864-016-2476-x (PMC4765147; doi:10.1186/s12864-016-2476-x)
Supplement: Additional file 7: — Primers used for cloning, PCR, RT-PCR and q RT-PCR. (DOCX 15 kb) [file 12864_2016_2476_MOESM7_ESM.docx]

**Primers for *Arabidopsis* insertion scanning:**

SALK_044701： CFP- 5’-TCATCGGGTGTCTTTTTGAAC-3’

CRP- 5’-CTTTTGAAGACTTTCGGGGAC-3’

SALK_013945： DFP - 5’-TTGACTAGCGGGAATGTGAAG-3’

DRP-5’-CGTAACGAGACACACATTGAGTC-3’

**Primers for insertion effect investigation by RT-PCR:**

SALK_044701： CRTF-5’-ATCCTTTGCATATACTTGTTC-3’

CRTR-5’-ACAAAGACATAGGCTGTG-3’；

SALK_013945： DRTF-5’-CAATAAGAGATTCGCAGACGG-3’

DRTR-5’-CGCGTCTTTTACGTTTTTGAC-3’

**Primers for gene *Mf3* RNAi segment cloning:**

KpnI-5’-TAGGTACCTGGGTTTATTCGCCTTGTGCTTTG-3’

BamHI-5’-TAGGATCCGTACCAAGCTGCTCTACATTGC-3’

SpeI-5’-TAACTAGTTGGGTTTATTCGCCTTGTGCTTTG-3’

SacI-5’-TAGAGCTCGTACCAAGCTGCTCTACATTGC-3’

**Primers for *q*RT-PCR of known inositol phosphate metabolism genes:**

IPTK-1R–5’-AGCAGCATTCGCCAGATACTACAAC-3’

IPTK-1F-5’-GCAACCAACAGGAGGAGAAAGG-3’

MIKR-5’-GGACTGCAGTGCTGGTTATTTTG-3’

MIKF-5’-CTGCGGTGCTGATGTCTGAAC-3’

ZmMRP4R-5’-CAGCAGCTGGTAGAAATTCAACTTG-3’

ZmMRP4F-5’-GCTTGATGATCCCTTCAGTGCTG-3’

GAPDHF-5’- CCCTTCATCACCACGGACTAC-3’

GAPDHR-5’- AACCTTCTTGGCACCACCCT-3’

**Primers for *q*RT-PCR of candidate genes:**

CB5(GRMZM2G075900)R-5’-TAAACACCTCATCTACAAGTCGC-3’

CB5(GRMZM2G075900)F-5’-AAAGGCAGTGAGTCCCATAAG-3’

DH2(GRMZM5G874955)R-5’-TTTGTCTCCTAGGAGGTGATTTC-3’

DH2(GRMZM5G874955)F-5’-CTACTTCGACTTCAAGCACAGC-3’

Mf3(GRMZM2G123544)F-5’- CGACCCTTGTGATGCTGAAACTG-3’

Mf3(GRMZM2G123544)R-5’- GGGTAGGTGAGTTCTTGGTTTTGC-3’

**Primers for GFP fusion construction (in pRTL2):**

CB5-GFP-XhoI-5’-CTCTCGAGATGAGGGCAAATTCATATCC-3’

CB5-GFP-SacI-5’-CTGAGCTCCAAGAATATGGGAG-3’

DH2-GFP-BamHI-5’-TGGATCCATGGTTGGTTTCACCATAC-3’

DH2-GFP-XbaI-5’-CTCTAGATCTCTTCTGGAAGTTAAGC-3’

Mf3-GFP-BamHI-5’-TGGATCCATGGCGGAAGAGGCATC

Mf3-GFP-SmaI-5’-TACCCGGGTGACGACGCGCAAGG
